# Supplementary material for: Temporal trends in heart failure medication prescription in a population-based cohort study
Source: BMJ Open. 2021 Mar 2;11(3):e043290. doi: 10.1136/bmjopen-2020-043290 (PMC7929882; doi:10.1136/bmjopen-2020-043290)
Supplement: Supplementary data [file bmjopen-2020-043290supp001.pdf]

Table S1 – additional READ codes used to identify heart failure in the Clinical Practi[illegible] Research Datalink

CPRD*<br><br>Heart Failure READ codes
585g.00, G5yyC00, G5yyA00, G583.12, G583.11, G583.00, G5yy900, 585f.00

Legend Table S1. * CPRD = Clinical Practice Research Datalink
