# Supplementary figures and images for: Temporal trends in heart failure medication prescription in a population-based cohort study
Source: BMJ Open. 2021 Mar 2;11(3):e043290. doi: 10.1136/bmjopen-2020-043290 (PMC7929882; doi:10.1136/bmjopen-2020-043290)

## Supplemental material

**Figure S1 - study flow diagram**

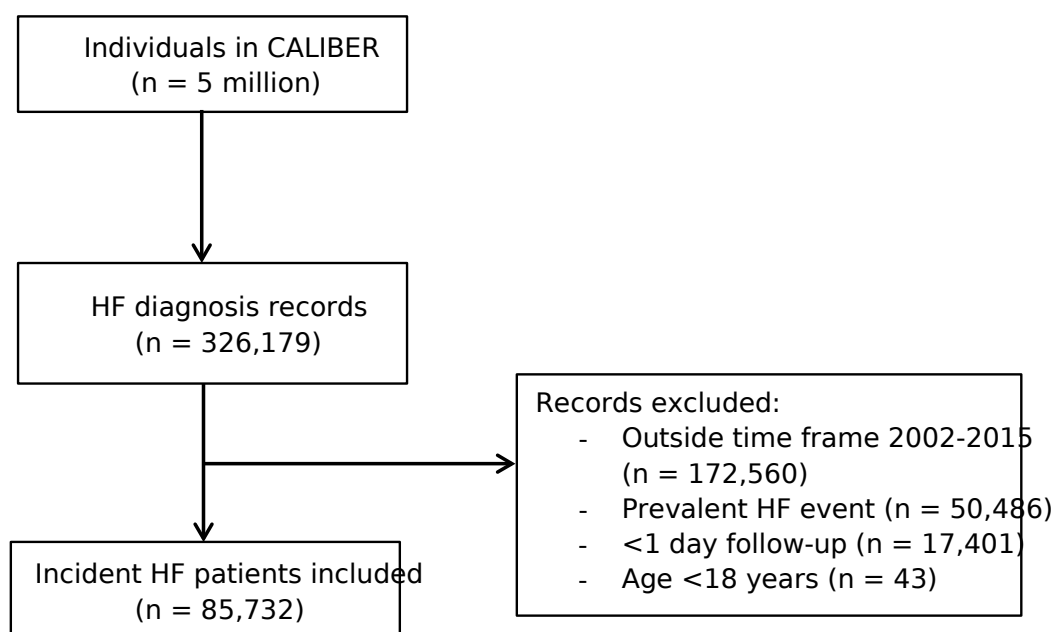

Supplement: Supplementary data [file bmjopen-2020-043290supp002.pdf]
