# Supplementary material for: Temporal trends in heart failure medication prescription in a population-based cohort study
Source: BMJ Open. 2021 Mar 2;11(3):e043290. doi: 10.1136/bmjopen-2020-043290 (PMC7929882; doi:10.1136/bmjopen-2020-043290)
Supplement: Author's manuscript [file bmjopen-2020-043290.draft_revisions.pdf]

BMJ Open

BMJ Open is committed to open peer review. As part of this commitment we make the peer review history of every article we publish publicly available.

When an article is published we post the peer reviewers' comments and the authors' responses online. We also post the versions of the paper that were used during peer review. These are the versions that the peer review comments apply to.

The versions of the paper that follow are the versions that were submitted during the peer review process. They are not the versions of record or the final published versions. They should not be cited or distributed as the published version of this manuscript.

BMJ Open is an open access journal and the full, final, typeset and author-corrected version of record of the manuscript is available on our site with no access controls, subscription charges or pay-per-view fees (http://bmjopen.bmj.com).

If you have any questions on BMJ Open's open peer review process please email info.bmjopen@bmj.com

BMJ Open

# Temporal Trends in Heart Failure Medication Use in a Population-Based Cohort Study



| | |
|---|---|
| Journal: | *BMJ Open* |
| Manuscript ID | bmjopen-2020-043290 |
| Article Type: | Original research |
| Date Submitted by the Author: | 05-Aug-2020 |



| | |
|---|---|
| Complete List of Authors: | Uijl, Alicia; UMC Utrecht, Julius Center for Health Sciences and Primary Care<br>Vaartjes, Ilonca; UMC Utrecht, Julius Center for Health Sciences and Primary Care<br>Denaxas, S; University College London, Institute for Health Informatics<br>Hemingway, Harry; University College London, Institute of Health Informatics<br>Shah, Anoop; University College London, Institute of Health Informatics<br>Cleland, J; Imperial College London, National Heart & Lung Institute<br>Grobbee, Diederick; UMC Utrecht, Julius Center for Health Sciences and Primary Care<br>Hoes, Arno; UMC Utrecht, Julius Center for Health Sciences and Primary Care<br>Asselbergs, Folkert; UMC Utrecht, Department of Cardiology; ICIN-Netherlands Heart Institute, Durrer Center for Cardiogenetic Research<br>Koudstaal, Stefan; UMC Utrecht, Department of Cardiology, Division Heart & Lungs |



| | |
|---|---|
| Keywords: | Heart failure < CARDIOLOGY, Cardiac Epidemiology < CARDIOLOGY, PUBLIC HEALTH |
| | |


SCHOLARONE™
Manuscripts

*I, the Submitting Author has the right to grant and does grant on behalf of all authors of the Work (as defined in the below author licence), an exclusive licence and/or a non-exclusive licence for contributions from authors who are: i) UK Crown employees; ii) where BMJ has agreed a CC-BY licence shall apply, and/or iii) in accordance with the terms applicable for US Federal Government officers or employees acting as part of their official duties; on a worldwide, perpetual, irrevocable, royalty-free basis to BMJ Publishing Group Ltd ("BMJ") its licensees and where the relevant Journal is co-owned by BMJ to the co-owners of the Journal, to publish the Work in this journal and any other BMJ products and to exploit all rights, as set out in our licence.*

*The Submitting Author accepts and understands that any supply made under these terms is made by BMJ to the Submitting Author unless you are acting as an employee on behalf of your employer or a postgraduate student of an affiliated institution which is paying any applicable article publishing charge ("APC") for Open Access articles. Where the Submitting Author wishes to make the Work available on an Open Access basis (and intends to pay the relevant APC), the terms of reuse of such Open Access shall be governed by a Creative Commons licence – details of these licences and which Creative Commons licence will apply to this Work are set out in our licence referred to above.*

*Other than as permitted in any relevant BMJ Author's Self Archiving Policies, I confirm this Work has not been accepted for publication elsewhere, is not being considered for publication elsewhere and does not duplicate material already published. I confirm all authors consent to publication of this Work and authorise the granting of this licence.*

For peer review only

**Title:** Temporal Trends in Heart Failure Medication Use in a Population-Based Cohort Study

**Authors:** Alicia Uijl PhD[1,2,3,4], Ilonca Vaartjes PhD[1], Spiros Denaxas PhD[3,4,5,6], Harry Hemingway MD PhD[3,4,6], Anoop D. Shah MD PhD[3,4], John Cleland MD PhD[7,8], Diederick E. Grobbee MD PhD[1], Arno W. Hoes MD PhD[1], Folkert W. Asselbergs MD PhD[3,4,9,10], Stefan Koudstaal MD PhD[3,4,9]

**Author affiliations:**

1. Julius Global Health, Julius Center for Health Sciences and Primary Care, University Medical Center Utrecht, Utrecht University, the Netherlands
2. Division of Cardiology, Department of Medicine, Karolinska Institutet, Stockholm, Sweden
3. Institute of Health Informatics, University College London, London, UK
4. Health Data Research UK, London, UK
5. The National Institute for Health Research University College London Hospitals Biomedical Research Centre, University College London, London, UK
6. Alan Turing institute, London, UK
7. Robertson Centre for Biostatistics & Clinical Trials, University of Glasgow, UK
8. National Heart & Lung Institute, Imperial College, London, UK
9. Department of Cardiology, Division Heart & Lungs, University Medical Center Utrecht, Utrecht University, the Netherlands
10. Institute of Cardiovascular Science, Faculty of Population Health Sciences, University College London, London, UK

**Word count**

Abstract: 236 words; Text, excluding references and figure legends: 2928 words.

**Correspondence**

Alicia Uijl (a.uijl@umcutrecht.nl)

Julius Center for Health Sciences and Primary Care, University Medical Center Utrecht

Heidelberglaan 100, 3508 GA Utrecht, the Netherlands



For peer review only - http://bmjopen.bmj.com/site/about/guidelines.xhtml

## Abstract

*Objective:* We examined temporal heart failure (HF) prescription patterns in a large representative sample of real-world patients in the UK, using electronic health records (EHR).

*Methods:* From the CALIBER resource, we identified 85,732 patients with a HF diagnosis between 2002-2015. Almost 50% of HF patients were women and the median age was 79.1 [70.2-85.7] years, with age at diagnosis increasing over time.

*Results:* We found several trends in pharmacological HF management, including increased beta-blocker prescriptions over time (29% in 2002-2005 and 54% in 2013-2015), which was not observed for mineralocorticoid receptor-antagonists (MR-antagonists) (18% in 2002-2005 and 18% in 2013-2015); higher prescription rates of loop diuretics in women and elderly patients together with lower prescription rates of RAS-inhibitors, beta-blockers, or MR-antagonists in these patients; little change in medication prescription rates after 6 months of HF diagnosis; and lastly, patients hospitalised for HF who had no follow-up in primary care had considerably lower prescription rates compared to patients with a HF diagnosis in primary care with or without HF hospitalisation.

*Conclusion:* In the general population, the use of MR-antagonists for HF remained low and did not change throughout 13 years of follow up. With large differences between HF patients, with lowest prescription rates observed in women, elderly patients, and those not followed-up in primary care, these findings suggest HF management can be improved by focusing effort and healthcare resources on improving communication between primary and secondary care.


**Keywords:** Prescription patterns, ACE-inhibitors, ARB, RAS-inhibitors, Beta-blockers, MRA, Loop diuretics, Heart failure, Electronic health records.



For peer review only - http://bmjopen.bmj.com/site/about/guidelines.xhtml

## Article summary

Strengths and limitations of the study

- Large cohort of HF patients from primary and secondary care
- Long follow-up period of almost 15 years
- Unable to differentiate between HF subphenotypes HFrEF or HFpEF
- Unknown treatment eligibility, contraindications or intolerances that may affect the choice of treatment

For peer review only



For peer review only - http://bmjopen.bmj.com/site/about/guidelines.xhtml

**Key questions**

What is already known about this subject?

Previous studies suggest that many heart failure (HF) patients do not receive guideline recommended therapies. Optimal treatment seems to be particularly challenging in elderly patients, women or patients with multiple comorbidities and contraindications for treatments

What does this study add?

This study shows the prescription trends in HF patients in the general population and medication use in HF patients in the years prior to their HF diagnosis.

How might this impact on clinical practice?

Our findings suggest that HF management could be improved by focusing healthcare resources on improving communication between primary and secondary care. We need to direct more effort towards effective implementation of guideline-recommended therapies in real-world HF care.



For peer review only - http://bmjopen.bmj.com/site/about/guidelines.xhtml

**Abbreviations**

| | |
|---|---|
| HF | Heart failure |
| EHR | Electronic health records |
| CPRD | Clinical Practice Research Datalink |
| HES | Hospital Episode Statistics |
| ONS | Office for National Statistics |
| COPD | Chronic obstructive pulmonary disease |
| RAS-inhibitors | Angiotensin converting enzyme-inhibitors and/or angiotensin II receptor blockers |
| MR-antagonists | Mineralocorticoid receptor-antagonists |
| HFrEF | Heart failure with reduced ejection fraction |
| HFpEF | Heart failure with preserved ejection fraction |

For peer review only



For peer review only - http://bmjopen.bmj.com/site/about/guidelines.xhtml

## Introduction

Heart failure (HF) is a common public health burden, with the prevalence of HF estimated at approximately 500.000 patients in the UK.(1, 2) Once diagnosed, initiation and up titration of guideline recommended therapies can reduce morbidity and mortality, however 5-year survival still remains 20% to 50%.(3, 4)

Several observational studies have assessed treatment uptake in HF patients following their diagnosis. These studies suggest that many patients did not receive guideline recommended therapies, or at low doses with sparse attempts for up titration.(5–8) Optimal treatment for effective disease management seems to be particularly challenging in elderly patients, women or patients with multiple comorbidities and contraindications for treatments.(7, 8) At present, few data are available for prescription trends in HF patients in the general population and even fewer data are available that shed light on medication use in HF patients in the years prior to their HF diagnosis.

The CALIBER resource curates primary and secondary care EHR of 5 million individuals in the UK, including HF diagnosis and medication prescriptions.(9) Given the amount of information available, medication use of all HF patients in the community may be investigated – including those which are underrepresented in heart failure disease registries of randomised clinical trials.

Therefore, we sought to examine HF treatment prescription patterns following a HF diagnosis for the overall population as well as specific subgroups based on gender (e.g. women), age (e.g. elderly), social economic status and healthcare setting (e.g. primary care or secondary care), in a large representative sample of real-world patients in the UK, using electronic health records (EHR). (10)



For peer review only - http://bmjopen.bmj.com/site/about/guidelines.xhtml

## Methods

*Data source*

Patients were selected from the CALIBER resource, which consists of three linked databases: The Clinical Practice Research Datalink (CPRD) with primary care EHR, Hospital Episodes Statistics (HES) containing coded diagnoses and surgical procedures from inpatient hospital admissions, and the Office for National Statistics (ONS) registry containing cause-specific mortality data.(10) Previous work has shown that these patients are representative of the general population in the UK.(11, 12)

*Study population*

Patients were included at their first record of HF from CPRD or HES between January 1st 2002 and December 31st 2015. In CPRD, events were defined by a diagnosis of HF based on READ clinical codes and in HES by a diagnosis of HF based on ICD-10 codes. The same HF diagnosis codes were used as in previous papers, with in addition several newer READ codes listed in **Table S1**.(13, 14) All patients were eligible for inclusion if they were aged 18 years or older, were registered with a GP for at least one year prior to diagnosis of HF, in a practice that had at least one year of up-to-standard data recording in CPRD. The first record of HF from CPRD or HES was considered the index date. Individuals were censored at the earliest date from the date of de-registration, the last data collection date, the date of death or at the study end date (31st December 2015). Data from HF patients up to 3 years prior to index date was included in this study.

*Patient and public involvement*

There was no patient or public involvement in this research.



For peer review only - http://bmjopen.bmj.com/site/about/guidelines.xhtml

*HR phenotyping variables*

Baseline patient characteristics were based on records from CPRD and/or HES prior to index date, including demographics (age, sex, ethnicity, social deprivation) cardiovascular risk factors (smoking, BMI, diastolic blood pressure and systolic blood pressure and estimated glomerular filtration rate, comorbidities (a medical history of atrial fibrillation, chronic obstructive pulmonary disease (COPD), diabetes, hypertension, ischaemic heart disease, valvular disease and history of cancer) and medication prescription, classified as: RAS-inhibitors (Angiotensin converting enzyme-inhibitors and/or angiotensin II receptor blockers), beta-blockers, mineralocorticoid receptor-antagonists (MR-antagonists) and loop diuretics. Definitions of these variables could be found online at http://www.caliberresearch.org/portal/.

Medication prescription for RAS-inhibitors, beta-blockers, MR-antagonists and loop diuretics was identified between three years prior to HF diagnosis up to three years after HF diagnosis per the following increments: -36 months to -24 months, -24 months to -18 months, -18 months to -12 months, -12 months to -6 months, -6 months to -3 months, -3 months to HF diagnosis, HF diagnosis to +3 months, +3 months to +6 months, +6 months to +12 months, +12 months to +18 months, +18 months to +24 months and +24 to +36 months.

Healthcare setting was characterised as primary care only (no HF hospitalisation), secondary care only (no HF diagnosis recorded in primary care) or HF diagnosis in both primary and secondary care. Ethnicity records from CPRD and HES were combined and categorised as Caucasian, Asian, Black or Other. Social deprivation was measured as quintiles of the index of multiple deprivation of the geographical area of the primary care practice, a score calculated based on seven indices of deprivation: income, employment, health and disability, education, barrier to housing and services, crime and living environment.(15) Smoking status was classified as never, ex- or current smokers.



For peer review only - http://bmjopen.bmj.com/site/about/guidelines.xhtml

*Statistical analysis*

Patient characteristics were summarised as mean (SD) or median [IQR] for continuous variables and percentages for categorical variables. The percentage of HF patients prescribed pharmacological treatments was calculated per increment and per time period as defined by publication year of previous ESC guidelines (2001, 2005, 2008 and 2012)(1, 16–19): 2002-2005, 2006-2008, 2009-2012 and 2013-2015. In addition to the overall cohort, we investigated several subgroups: age (< vs. ≥ 75 years old), sex (men vs. women), social economic status (lowest quintile of social deprivation vs. the rest) and setting (only follow-up in primary care vs. only in secondary care vs. follow-up in primary care after HF hospitalisation). All analyses were performed using R version 3.6.1.

## Results

*Baseline characteristics*

We identified 85,732 patients with a HF diagnosis. The study flow diagram could be found in **Figure S1**. Median follow-up after HF diagnosis (index date) was 2.1 years [0.6 – 4.5] years. **Table 1** shows the overall baseline patient characteristics and per time period 2002-2005, 2006-2008, 2009-2012 and 2013-2015. Almost 50% of patients were women and the median age was 79.1 [70.2 - 85.7] years, with age at HF diagnosis increasing over time. Overall, many HF patients had comorbidities, most common were hypertension (61%), ischaemic heart disease (44%) and atrial fibrillation (37%), with increasing numbers of patients with comorbidities over time. Approximately 40% (n= 34,489) of patients were followed-up in primary care after a HF hospitalisation, 20% (n= 15,330) of patients were only known in primary



For peer review only - http://bmjopen.bmj.com/site/about/guidelines.xhtml

care and never hospitalised for HF and the remaining 40% (n= 35,913) of patients had no follow-up in primary care after HF hospitalisation.

*Overall prescription patterns*

Overall prescription patterns are shown in **Figure 1**. Many patients were prescribed medication before HF diagnosis, especially RAS-inhibitors (20% in 2002-2005 to 46% in 2013-2015). Over time, beta-blocker prescription after HF diagnosis increased from 30% in 2002-2005 to 55% in 2013-2015. Throughout the follow up of 13 years, there were little observed changes for MR-antagonist uptake, this remained at 20% throughout time after HF diagnosis. The largest observed changes in prescription patterns occurred between 6 months before and after HF diagnosis (**Figure 1**). Approximately 20% of HF patients were prescribed a loop diuretic up to three years prior to HF diagnosis.

*Setting-specific prescription patterns*

Setting-specific prescription patterns are shown in **Figure 2**. Patients followed-up in primary care after HF hospitalisation had the highest prescription rates for all types of medication. Over time, the prescription for loop-diuretics, RAS-inhibitors and beta-blockers converged together. In these patients the prescription for MR-antagonists increased over time after HF diagnosis from 20% in 2002-2005 to 30% in 2013-2015.

Patients known in primary care but never hospitalised for HF had lower prescription rates for all types of treatment compared to patients with primary care follow-up and at least one HF hospitalisation. Mainly loop diuretics were less prescribed in these patients and the prescription of loop diuretics decreased over time with 65% of patients receiving loop diuretics after HF diagnosis in 2002-2005 compared to just over 40% in 2013-2015.



For peer review only - http://bmjopen.bmj.com/site/about/guidelines.xhtml

Patients hospitalised for HF but without a HF diagnosis in primary care, had the lowest prescriptions rates for loop diuretics, RAS-inhibitors and beta-blockers, which remained stable over time (50%, 45%, and 45% in 2013-2015 respectively). MR-antagonists were only prescribed in 13% of patients after HF diagnosis, this was similar for each time period.

*Age-specific prescription patterns*

Differences in prescription according to age categories are shown in **Figure 3**. The observed increase in prescriptions for RAS-inhibitors, beta-blockers, and MR-antagonists between 6 months before HF diagnosis to 6 months after HF diagnosis was less pronounced in elderly patients. The average increase in elderly patients was 12%, 7%, 8% for RAS-inhibitors, beta-blockers and MR-antagonists respectively, while younger patients had an average increase of 23%, 19% and 13% for RAS-inhibitors, beta-blockers and MR-antagonists respectively. On the other hand, a higher proportion of elderly patients were treated with loop-diuretics compared to younger patients, both before and after HF diagnosis (45% before and 63% after HF diagnosis in elderly compared to 27% before and 47% after HF diagnosis for younger patients in 2013-2015). After HF diagnosis, a higher percentage of younger patients were prescribed with RAS-inhibitors and beta-blockers compared to older patients.

*Sex-specific prescription patterns*

Differences in prescription between men and women are shown in **Figure 4**. Loop diuretics were prescribed in a higher proportion of women compared to men, this difference was already present prior to HF diagnosis where 6 months before diagnosis 30% of women and 20% of men were prescribed a loop diuretic. After HF diagnosis, the most prescribed medication for women was a loop diuretic, while a higher proportion of men were prescribed



For peer review only - http://bmjopen.bmj.com/site/about/guidelines.xhtml

a RAS-inhibitor. Men were also more often prescribed RAS-inhibitors, beta-blockers and MR-antagonists after HF diagnosis compared to women.

*Social economic status-specific prescription patterns*

Social economic status-specific prescription patterns are shown in **Figure 5**. We did not observe any discernible differences between patients in low vs. high social-economic areas (highest quintile of social economic deprivation).

## Discussion

In this large-scale study of 85,732 HF patients we investigated treatment prescription patterns in a representative sample of real-world patients with HF in the UK between 2002 and 2015. We found three important trends in pharmacological HF management: a) increased use of beta-blockers, whereas there was no increased uptake of MR-antagonists over 13 years follow up; b) prescription rates remained almost unchanged after the first 6 months following a HF diagnosis; and lastly, c) higher rates of loop diuretics in women and elderly patients together with lower prescription rates for RAS-inhibitors, beta-blockers, or MR-antagonists.

*Temporal trends in heart failure medication*

Even though prescription rates increased over time from 2002 to 2015, overall prescription rates remained low. This is in line with previously published studies.(5–8, 20) Low prescription rates could be attributed to the mixed HF cases found in EHR. We were unable to distinguish HF with reduced ejection fraction (HFrEF) and HF with preserved ejection fraction (HFpEF) based on medical records, thereby including known differences in treatment recommendations for these HF phenotypes.(1)



For peer review only - http://bmjopen.bmj.com/site/about/guidelines.xhtml

For peer review only

We found no major differences in prescription behaviour after the publication of ESC guidelines, however we did observe the gradual increase of beta-blockers as one of the cornerstones of HF treatment. RAS-inhibitors were prescribed in a high proportion of patients throughout the years of the study, presumably because the first clinical trials in HFrEF showing a beneficial effect were from the late 1980s and early 1990s.(21) Surprisingly, we found lower than expected prescription rates for MR-antagonists, which persisted over the years included in this study. This is in spite of multiple clinical trials which have shown benefit in HFrEF patients.(22) Besides HFrEF trials, a post-hoc analysis of the TOPCAT trial in 2015 (Spironolactone, a MR-antagonist, for HFpEF) reported regional differences between Americas and Russia/Georgia, where the American patients showed clinical benefits.(23) The American College of Cardiology/American Heart Association focused update on HF management in 2017 gave spironolactone a grade IIb recommendation, thereby stimulating that selected HFpEF patients could be treated with spironolactone to decrease re-hospitalisations.(24)

*Heart failure medication initiation following diagnosis*

Most activity in treatment prescription behaviour was observed between 6 months before to 6 months after HF diagnosis. After the 6 month mark we did not observe many patients starting any of the medication investigated. This is in line with previous studies showing that there are few changes in medication use and little up titration of medication after treatment initiation.(5, 25) This leaves room for improvement in starting treatment longer after HF diagnosis, especially as patients hospitalised with acute HF may not immediately tolerate negative inotropic medication such as beta-blockers.



For peer review only - http://bmjopen.bmj.com/site/about/guidelines.xhtml

*Impact of heart failure hospitalisation on medication prescription*

We found differences in prescription patterns between patients if with HF diagnosis recorded in different settings. Patients with a primary care HF diagnosis without HF hospitalisation had much lower prescription rates of loop diuretics compared to patients with a HF diagnosis recorded in both primary and secondary care. It could be that these patients have less severe fluid overload that requires alleviation by loop diuretics.

Previously it was shown that there are differences in overall five-year survival of patients with HF diagnosis recorded in primary care only, secondary care only and in both, with the worst survival seen in HF patients identified only in secondary care and the best survival for HF patients identified in primary care with or without hospitalisation for HF.(13) Here, we advance current knowledge by showing that there are longitudinal differences in HF care of patients with diagnosis recorded in different settings. Importantly, HF patients with HF hospitalisation and no diagnosis of HF recorded in primary care had the lowest prescription rates, signifying a potential quality of care gap between secondary and primary care, where patients are not treated optimally. Primary care is the basis of many healthcare systems, including the UK. If there is no HF diagnosis recorded in primary care after HF hospitalisation, which is indicative for worse survival, rehospitalisation and severity of disease, this could be detrimental for patients.

For peer review only

*Heart failure treatment in women and elderly*

Over time, we observed that HF was diagnosed at a later age, with the median almost 80 years old between 2013-2015. This is also seen in many other developed countries where the mean age of HF diagnosis is over 70 years old.(26, 27)



For peer review only - http://bmjopen.bmj.com/site/about/guidelines.xhtml

We observed lower prescription rates in elderly patients compared to younger patients for RAS-inhibitors, beta-blockers and MR-antagonists, although the difference in MR-antagonists was less pronounced. Many elderly patients were already using RAS-inhibitors prior to HF diagnosis, therefore the increase in prescription rate is not as steep as compared to younger HF patients who are prescribed less medication prior to HF diagnosis. This could be explained by the presence of comorbidities, such as atrial fibrillation or hypertension, which are much more prevalent among elderly compared to younger patients, and for which these elderly patients could be prescribed RAS-inhibitors.

Remarkably, the difference between prescription of RAS-inhibitors and beta-blockers prior to HF diagnosis was less than 5% for men and women, and only after the diagnosis of HF was a higher proportion of men prescribed a RAS-inhibitor or beta-blocker. This could potentially be related to the fact that elderly women are more likely to develop HFpEF and therefore tend to be treated symptomatically with loop diuretics, rather than with RAS-inhibitors and beta-blockers. However, the literature also shows that there are differences in treatment prescription in men and women with HFrEF, for which there is no obvious explanation.(28)

Both elderly patients and women received more loop diuretics. However, this could potentially be harmful, especially for elderly, since loop diuretics could lead to electrolyte disturbances and acute kidney injury.(29) Elderly patients are often excluded or underrepresented in clinical trials, therefore current recommendations lack convincing evidence in the elderly population. However, recently a large meta-analysis reported a significant effect of beta-blockers on overall mortality regardless of age.(30) These studies indicate that elderly patients also benefit from HF-specific medication and should be a choice of treatment for these patients, besides loop diuretics for symptom alleviation.



For peer review only - http://bmjopen.bmj.com/site/about/guidelines.xhtml

*Strengths and limitations*

Strengths of this study are the large cohort of HF patients and a long follow-up period. Patient records available are representative of the general UK population, which provides evidence for the validity of using these EHR for research.(11, 12) However, we were limited by the inability to differentiate between HF phenotypes based on medical records, since there was no access to detailed echocardiography estimates to assess systolic function. We were also unable to assess patients' symptom class (which would affect their eligibility for treatments such as MRA-antagonists), and contraindications or intolerances that may affect the choice of medication.

*Conclusion*

The results of this population-based study of over 80,000 patients with heart failure in England shows variable increases in uptake of evidence-based treatments, with no change in prescription of MR-antagonists over 13 years, but an increase in beta-blocker use. Large differences were observed between HF patient groups, with lowest prescription rates in women, elderly patients, and those without a primary care diagnosis. These findings suggest HF management can be improved by focusing effort and healthcare resources on improving communication between primary and secondary care. There is still a need for effective implementation of guideline-recommended therapies in real-world HF care.



For peer review only - http://bmjopen.bmj.com/site/about/guidelines.xhtml

**Acknowledgements**

This study was approved by the Medicines and Healthcare Products Regulatory Agency Independent Scientific Advisory Committee protocol reference: 17_015. This study is based in part on data from the Clinical Practice Research Datalink obtained under license from the UK Medicines and Healthcare products Regulatory Agency. The data are provided by patients and collected by the NHS as part of their care and support. The interpretation and conclusions contained in this study are those of the author(s) alone. Hospital Episode Statistics Copyright (2019) is reused with the permission of The Health & Social Care Information Centre. All rights reserved.

**a. Contributor statement**

AU has designed the research, analysed and interpreted the data and drafted the manuscript. IV, AH, FA and SK have designed the research, interpreted the data, critically revised the manuscript and supervised AU. SD, HH, AS, JC and DG have interpreted the data and critically revised the manuscript.

**b. Competing interests**

The authors report no conflict of interest.

**c. Funding**

This study is part of the BigData@Heart program that has received funding from the Innovative Medicines Initiative 2 Joint Undertaking under grant agreement No 116074. This Joint Undertaking receives support from the European Union's Horizon 2020 research and innovation programme and EFPIA.



For peer review only - http://bmjopen.bmj.com/site/about/guidelines.xhtml

This work was supported by Health Data Research UK, which receives its funding from Health Data Research UK Ltd (NIWA1) funded by the UK Medical Research Council, Engineering and Physical Sciences Research Council, Economic and Social Research Council, Department of Health and Social Care (England), Chief Scientist Office of the Scottish Government Health and Social Care Directorates, Health and Social Care Research and Development Division (Welsh Government), Public Health Agency (Northern Ireland), British Heart Foundation, and the Wellcome Trust. Work at the University College London Institute of Health Informatics and Institute of Cardiovascular Science is supported by a British Heart Foundation Accelerator Award (AA/18/6/24223).

**FA** is supported by UCL Hospitals NIHR Biomedical Research Centre. **IV** is supported by a grant from the Dutch Heart Foundation [grant DHF project 'Facts and Figures']. **SD** is supported by an Alan Turing Fellowship. **HH** is supported by an NIHR Senior Investigator Award. **ADS** is funded by a post-doctoral fellowship from THIS Institute. **JC** received research grants from Bayer, Novartis and Vifor and honoraria for steering committees from Amgen, Bayer, Novartis and Servier.

### d. Data sharing statement

No additional data available.

For peer review only

## References

1. Ponikowski P, Voors AA, Anker SD, et al. 2016 ESC Guidelines for the diagnosis and treatment of acute and chronic heart failure: The Task Force for the diagnosis and treatment of acute and chronic heart failure of the European Society of Cardiology (ESC). *Eur. J. Heart Fail.* 2016;18:891–975.

2. British Heart Foundation Centre on Population Approaches for Non-Communicable Disease Prevention. British Heart Foundation Cardiovascular Disease Statistics 2015. 2015.

3. Bleumink GS, Knetsch AM, Sturkenboom MCJM, et al. Quantifying the heart failure epidemic: prevalence, incidence rate, lifetime risk and prognosis of heart failure The Rotterdam Study. *Eur. Heart J.* 2004;25:1614–1619.

4. Koudstaal S, Pujades-Rodriguez M, Denaxas S, et al. Prognostic burden of heart failure recorded in primary care, acute hospital admissions, or both: a population-based linked electronic health record cohort study in 2.1 million people. *Eur. J. Heart Fail.* 2016.

5. Deschaseaux C, McSharry M, Hudson E, Agrawal R, Turner SJ. Treatment Initiation Patterns, Modifications, and Medication Adherence Among Newly Diagnosed Heart Failure Patients: A Retrospective Claims Database Analysis. *J. Manag. care Spec. Pharm.* 2016;22:561–571.

6. Bress AP, King JB, Brixner D, et al. Pharmacotherapy Treatment Patterns, Outcomes, and Health Resource Utilization Among Patients with Heart Failure with Reduced Ejection Fraction at a U.S. Academic Medical Center. *Pharmacotherapy* 2016;36:174–186.

7. Stork S, Handrock R, Jacob J, et al. Treatment of chronic heart failure in Germany: a retrospective database study. *Clin. Res. Cardiol.* 2017.

8. Chin KL, Skiba M, Tonkin A, et al. The treatment gap in patients with chronic systolic heart failure: a systematic review of evidence-based prescribing in practice. *Heart Fail. Rev.* 2016;21:675–697.



For peer review only - http://bmjopen.bmj.com/site/about/guidelines.xhtml

9. Denaxas S, Gonzalez-Izquierdo A, Direk K, et al. UK phenomics platform for developing and validating electronic health record phenotypes: CALIBER. *J. Am. Med. Inform. Assoc.* 2019.

10. Denaxas SC, George J, Herrett E, et al. Data resource profile: cardiovascular disease research using linked bespoke studies and electronic health records (CALIBER). *Int. J. Epidemiol.* 2012;41:1625–1638.

11. Herrett E, Thomas SL, Schoonen WM, Smeeth L, Hall AJ. Validation and validity of diagnoses in the General Practice Research Database: a systematic review. *Br. J. Clin. Pharmacol.* 2010;69:4–14.

12. Burns EM, Rigby E, Mamidanna R, et al. Systematic review of discharge coding accuracy. *J. Public Health*. 2012;34:138–148.

13. Koudstaal S, Pujades-Rodriguez M, Denaxas S, et al. Prognostic burden of heart failure recorded in primary care, acute hospital admissions, or both: a population-based linked electronic health record cohort study in 2.1 million people. *Eur. J. Heart Fail.* 2017;19.

14. Uijl A, Koudstaal S, Direk K, et al. Risk factors for incident heart failure in age- and sex-specific strata: a population-based cohort using linked electronic health records. *Eur. J. Heart Fail.* 2019.

15. McLennan D, Barnes H, Noble M, Davies J, Garratt E. The English indices of deprivation, 2010: Technical Report. London: HMSO. 2011.

16. Remme WJ, Swedberg K. Guidelines for the diagnosis and treatment of chronic heart failure. *Eur. Heart J.* 2001;22:1527–1560.

17. Swedberg K, Drexler H, Follath F, et al. Guidelines for the diagnosis and treatment of chronic heart failure : executive summary ( update 2005 ) The Task Force for the Diagnosis and Treatment of Chronic Heart Failure of the European Society of Cardiology. *Eur. Heart J.* 2005:1115–1140.



For peer review only - http://bmjopen.bmj.com/site/about/guidelines.xhtml

18. Dickstein K, Cohen-Solal A, Filippatos G, et al. ESC guidelines for the diagnosis and treatment of acute and chronic heart failure 2008: the Task Force for the diagnosis and treatment of acute and chronic heart failure 2008 of the European Society of Cardiology. *Eur. J. Heart Fail.* 2008;10:933–989.

19. McMurray JJV, Adamopoulos S, Anker SD, et al. ESC Guidelines for the diagnosis and treatment of acute and chronic heart failure 2012: The Task Force for the Diagnosis and Treatment of Acute and Chronic Heart Failure 2012 of the European Society of Cardiology. *Eur. Heart J.* 2012;33:1787–1847.

20. Peters-Klimm F, Muller-Tasch T, Schellberg D, et al. Guideline adherence for pharmacotherapy of chronic systolic heart failure in general practice: a closer look on evidence-based therapy. *Clin. Res. Cardiol.* 2008;97:244–252.

21. SOLVD Investigators, Yusuf S, Pitt B, Davis CE, Hood WB, Cohn JN. Effect of enalapril on survival in patients with reduced left ventricular ejection fractions and congestive heart failure. *N. Engl. J. Med.* 1991;325:293–302.

22. Pitt B, Zannad F, Remme WJ, et al. The Effect of Spironolactone on Morbidity and Mortality in Patients with Severe Heart Failure. *N. Engl. J. Med.* 1999;341:709–717.

23. Pfeffer MA, Claggett B, Assmann SF, et al. Regional Variation in Patients and Outcomes in the Treatment of Preserved Cardiac Function Heart Failure With an Aldosterone Antagonist (TOPCAT) Trial. *Circulation* 2015;131:34–42.

24. Yancy CW, Jessup M, Bozkurt B, et al. 2017 ACC/AHA/HFSA Focused Update of the 2013 ACCF/AHA Guideline for the Management of Heart Failure: A Report of the American College of Cardiology/American Heart Association Task Force on Clinical Practice Guidelines and the Heart Failure Society of Amer. *J. Am. Coll. Cardiol.* 2017;68:1476–1488.

25. Greene SJ, Fonarow GC, DeVore AD, et al. Titration of Medical Therapy for Heart Failure



For peer review only - http://bmjopen.bmj.com/site/about/guidelines.xhtml

With Reduced Ejection Fraction. *J. Am. Coll. Cardiol.* 2019;73:2365–2383.

26. Brugts JJ, Linssen GCM, Hoes AW, Brunner-La Rocca HP. Real-world heart failure management in 10,910 patients with chronic heart failure in the Netherlands. *Netherlands Hear. J.* 2018;26:272–279.

27. Savarese G, Vasko P, Jonsson Å, Edner M, Dahlström U, Lund LH. The Swedish Heart Failure Registry: a living, ongoing quality assurance and research in heart failure. *Ups. J. Med. Sci.* 2019;124:65–69.

28. Dewan P, Rørth R, Jhund PS, et al. Differential Impact of Heart Failure With Reduced Ejection Fraction on Men and Women. *J. Am. Coll. Cardiol.* 2019;73:29–40.

29. Täger T, Fröhlich H, Seiz M, Katus HA, Frankenstein L. READY: relative efficacy of loop diuretics in patients with chronic systolic heart failure—a systematic review and network meta-analysis of randomised trials. *Heart Fail. Rev.* 2019.

30. Kotecha D, Manzano L, Krum H, et al. Effect of age and sex on efficacy and tolerability of beta blockers in patients with heart failure with reduced ejection fraction: individual patient data meta-analysis. *BMJ* 2016;353:i1855.

For peer review only



For peer review only - http://bmjopen.bmj.com/site/about/guidelines.xhtml

## Figure legends

### Central illustration Figure 1

**Legend Figure 1.** The percentage of HF patients receiving prescriptions of RAS-inhibitors, beta-blockers, MR-antagonists, loop diuretics per months since HF diagnosis. RAS-inhibitors = ACE-inhibitors and/or ARB; MR-antagonists = mineralocorticoid receptor antagonists.

**Legend Figure 2.** The percentage of HF patients receiving prescriptions of RAS-inhibitors, beta-blockers and MRAs per months since HF diagnosis, stratified by setting (primary care only, secondary care only, both primary and secondary care). RAS-inhibitors = ACE-inhibitors and/or ARB; MR-antagonists = mineralocorticoid receptor antagonists.

**Legend Figure 3.** The percentage of HF patients receiving prescriptions of RAS-inhibitors, beta-blockers and MRAs per months since HF diagnosis, stratified by age. RAS-inhibitors = ACE-inhibitors and/or ARB; MR-antagonists = mineralocorticoid receptor antagonists.

**Legend Figure 4.** The percentage of HF patients receiving prescriptions of RAS-inhibitors, beta-blockers and MRAs per months since HF diagnosis, stratified by sex. RAS-inhibitors = ACE-inhibitors and/or ARB; MR-antagonists = mineralocorticoid receptor antagonists.

**Legend Figure 5.** The percentage of HF patients receiving prescriptions of RAS-inhibitors, beta-blockers and MRAs per months since HF diagnosis, stratified by social status (highest quintile of social deprivation vs. the rest). RAS-inhibitors = ACE-inhibitors and/or ARB; MR-antagonists = mineralocorticoid receptor antagonists.

For peer review only

**Table 1. Patients characteristics of heart failure patients between 2002 and 2015**

| | Overall | 2002 - 2005 | 2006 - 2008 | 2009 - 2012 | 2013 - 2015 | % missing |
|---|---|---|---|---|---|---|
| n | 85732 | 25366 | 17715 | 26114 | 16537 | |
| **Demographics** | | | | | | |
| Age (Years, median [IQR]) | 79.1 [70.2, 85.7] | 78.7 [70.7, 84.9] | 78.7 [ 69.9, 85.4] | 79.5 [70.1, 86.3] | 79.7 [70.0, 86.4] | 0 |
| Sex (% Women) | 48.6 | 49.3 | 48.4 | 48.4 | 48.0 | 0 |
| Ethnicity (% Caucasian) | 96.5 | 97.5 | 96.9 | 96.1 | 95.1 | 3.5 |
| Social deprivation (% lowest quintile) | 24.3 | 25.1 | 25.0 | 24.0 | 22.9 | 0 |
| **Clinical and lifestyle measurements** | | | | | | |
| SBP (mmHg, mean (sd)) | 136.2 (20.7) | 140.6 (22.3) | 135.9 (20.7) | 134.6 (20.0) | 132.9 (18.7) | 13.0 |
| DBP (mmHg, mean (sd)) | 76.2 (12.0) | 78.4 (12.0) | 76.2 (12.0) | 75.4 (12.0) | 74.4 (11.6) | 13.0 |
| BMI (kg/m$^2$, mean (sd)) | 28.6 (6.6) | 28.2 (6.4) | 28.4 (6.6) | 28.7 (6.8) | 28.8 (6.8) | 54.0 |
| eGFR (min/m2/1.73mL, median [IQR]) | 58.4 [ 45.3, 72.1] | 54.7 [43.4, 66.1] | 56.5 [44.3, 68.8] | 60.5 [46.3, 75.3] | 62.9 [47.5, 78.2] | 24.0 |
| Smoking status (% Current) | 20.8 | 22.3 | 20.0 | 20.4 | 20.5 | 38.7 |
| **Medical history (%)**[¶] | | | | | | |
| Atrial Fibrillation | 36.6 | 28.4 | 36.3 | 40.6 | 43.0 | - |
| COPD | 17.9 | 14.8 | 17.3 | 19.5 | 21.0 | - |
| Diabetes | 22.3 | 18.1 | 22.2 | 23.7 | 26.7 | - |
| Hypertension | 60.7 | 46.0 | 60.7 | 67.9 | 72.0 | - |
| Ischaemic heart disease | 44.2 | 39.0 | 46.0 | 46.4 | 46.8 | - |
| Valvular disease | 16.5 | 9.5 | 14.9 | 19.9 | 23.8 | - |
| **Medication prescription up to 3 months after HF diagnosis (%)**[¶] | | | | | | |
| RAS-inhibitors | 60.8 | 59.6 | 63.5 | 62.0 | 57.6 | - |
| Beta-blockers | 42.5 | 28.9 | 41.0 | 49.3 | 54.1 | - |
| MR-antagonists | 18.0 | 18.4 | 17.9 | 17.6 | 18.2 | - |
| Loop diuretics | 63.0 | 68.4 | 63.5 | 61.1 | 57.0 | - |

For peer review only

**Legend Table 1.** * Assessed by index of multiple deprivation, § denotes prior medical history of given comorbidity, ¤ Medication 12 months prior to index date, Mean (SD) = Mean (Standard deviation), Median [IQR] = Median [Interquartile range], CPRD = Clinical Practice Research Datalink, SBP = systolic blood pressure, DBP = Diastolic blood pressure, BMI = Body Mass Index, eGFR = estimated glomerular filtration rate, COPD = Chronic Obstructive Pulmonary Disease, RAS-inhibitors = ACE-inhibitors and/or angiotensin II receptor blockers, MR-antagonists = mineralocorticoid receptor antagonist. ¶Medical conditions and prescriptions were considered absent if not recorded.

For peer review only



For peer review only - http://bmjopen.bmj.com/site/about/guidelines.xhtml

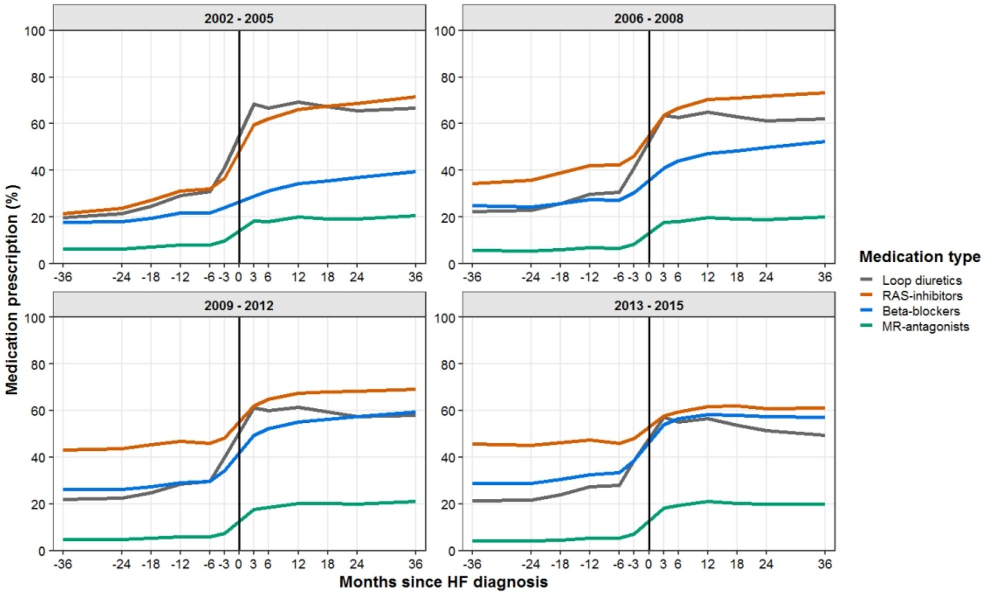


figure 1

127x76mm (300 x 300 DPI)

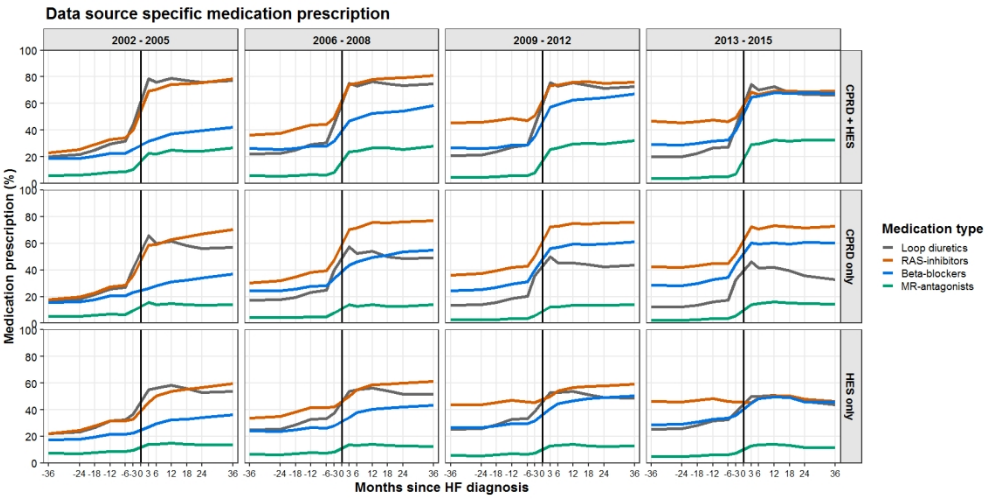


figure 2

152x76mm (300 x 300 DPI)

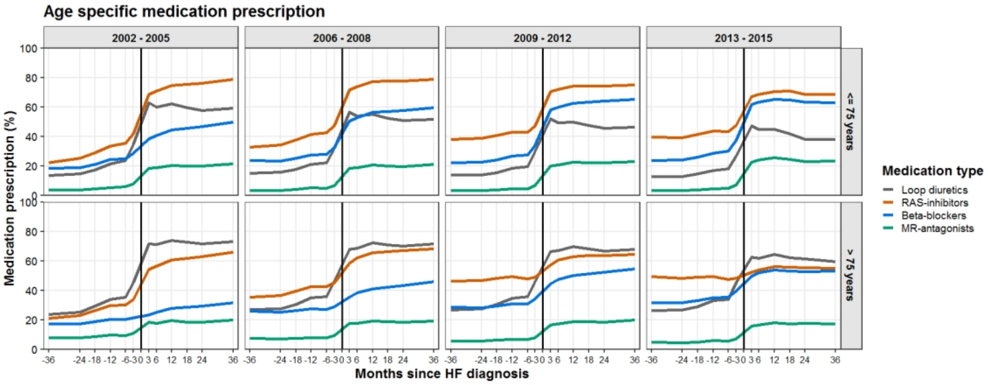


figure 3

152x59mm (300 x 300 DPI)

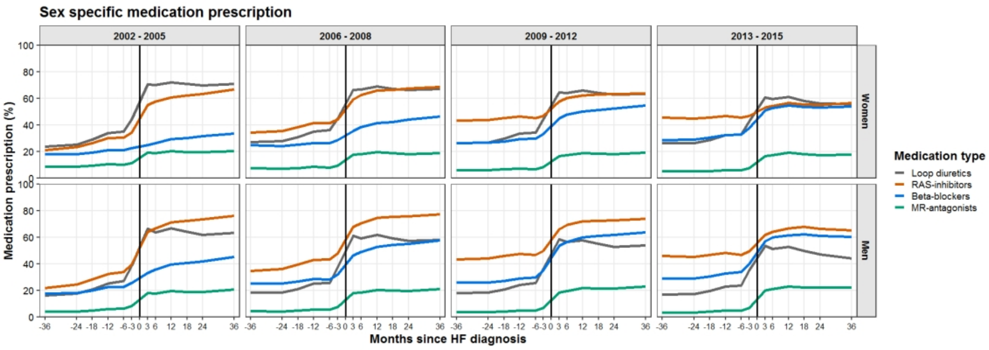


figure 4

169x59mm (300 x 300 DPI)

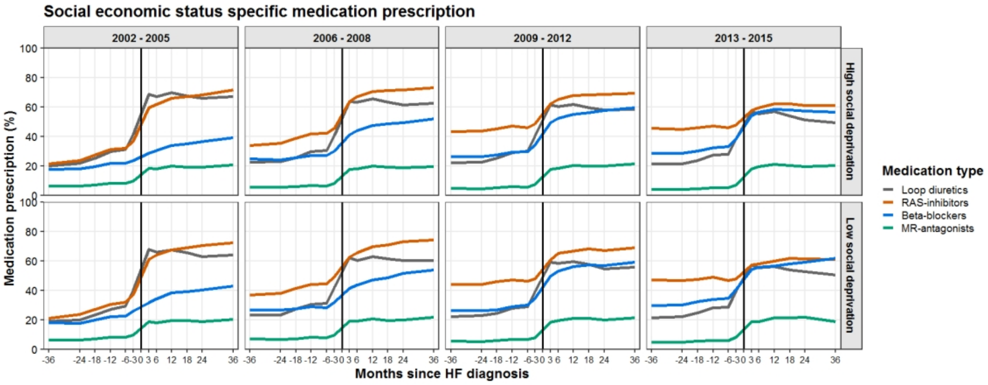


figure 5

152x59mm (300 x 300 DPI)

**Supplemental material**

**Figure S1 – study flow diagram**

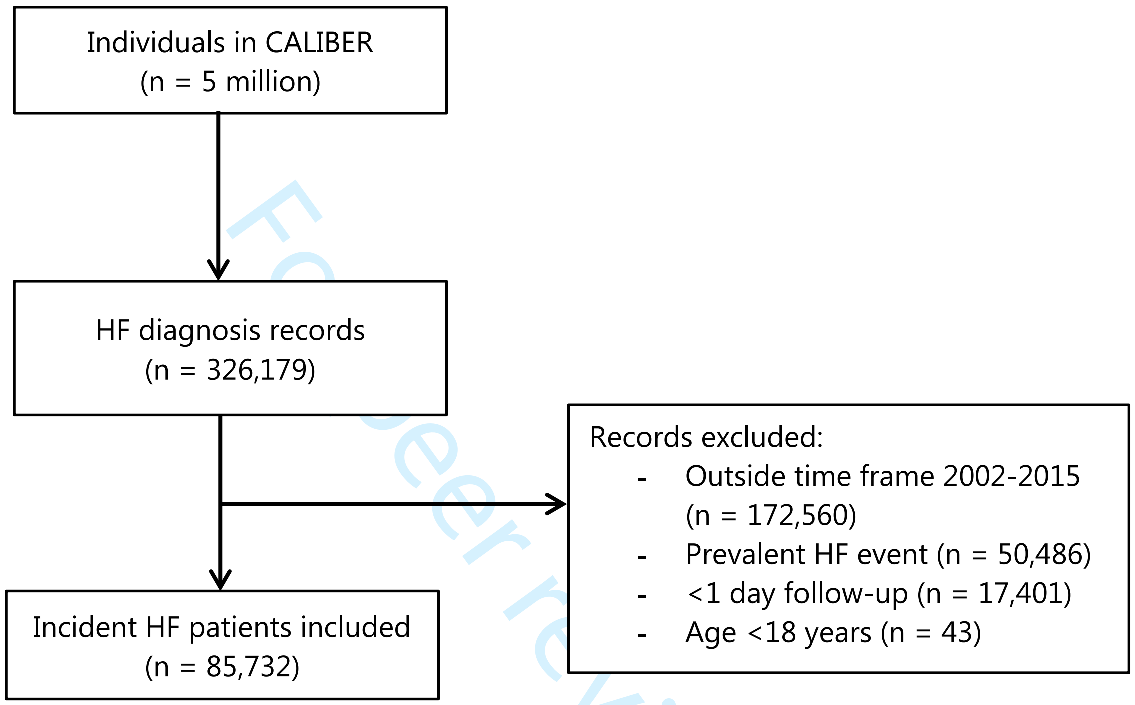


For peer review only

**Table S1 – additional READ codes used to identify heart failure in the Clinical Practice Research Datalink**

| CPRD* Heart Failure READ codes |
| --- |
| 585g.00, G5yyC00, G5yyA00, G583.12, G583.11, G583.00, G5yy900, 585f.00 |

**Legend Table S1**. * CPRD = Clinical Practice Research Datalink

For peer review only

STROBE Statement—Checklist of items that should be included in reports of ***cohort studies***

| | Item No | Recommendation | Page No |
|---|---|---|---|
| **Title and abstract** | 1 | (*a*) Indicate the study's design with a commonly used term in the title or the abstract<br>(*b*) Provide in the abstract an informative and balanced summary of what was done and what was found | 1, 3 |
| **Introduction** | | | |
| Background/rationale | 2 | Explain the scientific background and rationale for the investigation being reported | 6 |
| Objectives | 3 | State specific objectives, including any prespecified hypotheses | 6 |
| **Methods** | | | |
| Study design | 4 | Present key elements of study design early in the paper | 7 |
| Setting | 5 | Describe the setting, locations, and relevant dates, including periods of recruitment, exposure, follow-up, and data collection | 7 |
| Participants | 6 | (*a*) Give the eligibility criteria, and the sources and methods of selection of participants. Describe methods of follow-up<br>(*b*) For matched studies, give matching criteria and number of exposed and unexposed | 7 |
| Variables | 7 | Clearly define all outcomes, exposures, predictors, potential confounders, and effect modifiers. Give diagnostic criteria, if applicable | 8 |
| Data sources/ measurement | 8* | For each variable of interest, give sources of data and details of methods of assessment (measurement). Describe comparability of assessment methods if there is more than one group | - |
| Bias | 9 | Describe any efforts to address potential sources of bias | - |
| Study size | 10 | Explain how the study size was arrived at | - |
| Quantitative variables | 11 | Explain how quantitative variables were handled in the analyses. If applicable, describe which groupings were chosen and why | - |
| Statistical methods | 12 | (*a*) Describe all statistical methods, including those used to control for confounding<br>(*b*) Describe any methods used to examine subgroups and interactions<br>(*c*) Explain how missing data were addressed<br>(*d*) If applicable, explain how loss to follow-up was addressed<br>(*e*) Describe any sensitivity analyses | 9 |
| **Results** | | | |
| Participants | 13* | (a) Report numbers of individuals at each stage of study—eg numbers potentially eligible, examined for eligibility, confirmed eligible, included in the study, completing follow-up, and analysed<br>(b) Give reasons for non-participation at each stage<br>(c) Consider use of a flow diagram | 9 |
| Descriptive data | 14* | (a) Give characteristics of study participants (eg demographic, clinical, social) and information on exposures and potential confounders<br>(b) Indicate number of participants with missing data for each variable of interest<br>(c) Summarise follow-up time (eg, average and total amount) | 9 - 10 |
| Outcome data | 15* | Report numbers of outcome events or summary measures over time | 10 - 12 |

For peer review only



For peer review only - http://bmjopen.bmj.com/site/about/guidelines.xhtml

| | | | |
|---|---|---|---|
| Main results | 16 | (*a*) Give unadjusted estimates and, if applicable, confounder-adjusted estimates and their precision (eg, 95% confidence interval). Make clear which confounders were adjusted for and why they were included<br>(*b*) Report category boundaries when continuous variables were categorized<br>(*c*) If relevant, consider translating estimates of relative risk into absolute risk for a meaningful time period | 10 - 12 |
| Other analyses | 17 | Report other analyses done—eg analyses of subgroups and interactions, and sensitivity analyses | - |
| **Discussion** | | | |
| Key results | 18 | Summarise key results with reference to study objectives | 12 |
| Limitations | 19 | Discuss limitations of the study, taking into account sources of potential bias or imprecision. Discuss both direction and magnitude of any potential bias | 16 |
| Interpretation | 20 | Give a cautious overall interpretation of results considering objectives, limitations, multiplicity of analyses, results from similar studies, and other relevant evidence | 13 - 15 |
| Generalisability | 21 | Discuss the generalisability (external validity) of the study results | 13 - 15 |
| **Other information** | | | |
| Funding | 22 | Give the source of funding and the role of the funders for the present study and, if applicable, for the original study on which the present article is based | 2 |

*Give information separately for exposed and unexposed groups.

**Note:** An Explanation and Elaboration article discusses each checklist item and gives methodological background and published examples of transparent reporting. The STROBE checklist is best used in conjunction with this article (freely available on the Web sites of PLoS Medicine at http://www.plosmedicine.org/, Annals of Internal Medicine at http://www.annals.org/, and Epidemiology at http://www.epidem.com/). Information on the STROBE Initiative is available at http://www.strobe-statement.org.

For peer review only

BMJ Open

# Temporal Trends in Heart Failure Medication Prescription in a Population-Based Cohort Study



| | |
|---|---|
| Journal: | *BMJ Open* |
| Manuscript ID | bmjopen-2020-043290.R1 |
| Article Type: | Original research |
| Date Submitted by the Author: | 18-Nov-2020 |





| | |
|---|---|
| Complete List of Authors: | Uijl, Alicia; UMC Utrecht, Julius Center for Health Sciences and Primary Care<br>Vaartjes, Ilonca; UMC Utrecht, Julius Center for Health Sciences and Primary Care<br>Denaxas, S; University College London, Institute for Health Informatics<br>Hemingway, Harry; University College London, Institute of Health Informatics<br>Shah, Anoop; University College London, Institute of Health Informatics<br>Cleland, J; Imperial College London, National Heart & Lung Institute<br>Grobbee, Diederick; UMC Utrecht, Julius Center for Health Sciences and Primary Care<br>Hoes, Arno; UMC Utrecht, Julius Center for Health Sciences and Primary Care<br>Asselbergs, Folkert; UMC Utrecht, Department of Cardiology; ICIN-Netherlands Heart Institute, Durrer Center for Cardiogenetic Research<br>Koudstaal, Stefan; UMC Utrecht, Department of Cardiology, Division Heart & Lungs |





| | |
|---|---|
| <b>Primary Subject Heading</b>: | Epidemiology |
| Secondary Subject Heading: | General practice / Family practice, Cardiovascular medicine, Public health |
| Keywords: | Heart failure < CARDIOLOGY, Cardiac Epidemiology < CARDIOLOGY, PUBLIC HEALTH |
| | |

SCHOLARONE™
Manuscripts

*I, the Submitting Author has the right to grant and does grant on behalf of all authors of the Work (as defined in the below author licence), an exclusive licence and/or a non-exclusive licence for contributions from authors who are: i) UK Crown employees; ii) where BMJ has agreed a CC-BY licence shall apply, and/or iii) in accordance with the terms applicable for US Federal Government officers or employees acting as part of their official duties; on a worldwide, perpetual, irrevocable, royalty-free basis to BMJ Publishing Group Ltd ("BMJ") its licensees and where the relevant Journal is co-owned by BMJ to the co-owners of the Journal, to publish the Work in this journal and any other BMJ products and to exploit all rights, as set out in our licence.*

*The Submitting Author accepts and understands that any supply made under these terms is made by BMJ to the Submitting Author unless you are acting as an employee on behalf of your employer or a postgraduate student of an affiliated institution which is paying any applicable article publishing charge ("APC") for Open Access articles. Where the Submitting Author wishes to make the Work available on an Open Access basis (and intends to pay the relevant APC), the terms of reuse of such Open Access shall be governed by a Creative Commons licence – details of these licences and which Creative Commons licence will apply to this Work are set out in our licence referred to above.*

*Other than as permitted in any relevant BMJ Author's Self Archiving Policies, I confirm this Work has not been accepted for publication elsewhere, is not being considered for publication elsewhere and does not duplicate material already published. I confirm all authors consent to publication of this Work and authorise the granting of this licence.*

For peer review only

**Title:** Temporal Trends in Heart Failure Medication Prescription in a Population-Based Cohort Study

**Authors:** Alicia Uijl PhD[1,2,3,4], Ilonca Vaartjes PhD[1], Spiros Denaxas PhD[3,4,5,6], Harry Hemingway MD PhD[3,4,6], Anoop D. Shah MD PhD[3,4], John Cleland MD PhD[7,8], Diederick E. Grobbee MD PhD[1], Arno W. Hoes MD PhD[1], Folkert W. Asselbergs MD PhD[3,4,9,10], Stefan Koudstaal MD PhD[3,4,9]

**Author affiliations:**

1. Julius Global Health, Julius Center for Health Sciences and Primary Care, University Medical Center Utrecht, Utrecht University, the Netherlands
2. Division of Cardiology, Department of Medicine, Karolinska Institutet, Stockholm, Sweden
3. Institute of Health Informatics, University College London, London, UK
4. Health Data Research UK, London, UK
5. The National Institute for Health Research University College London Hospitals Biomedical Research Centre, University College London, London, UK
6. Alan Turing institute, London, UK
7. Robertson Centre for Biostatistics & Clinical Trials, University of Glasgow, UK
8. National Heart & Lung Institute, Imperial College, London, UK
9. Department of Cardiology, Division Heart & Lungs, University Medical Center Utrecht, Utrecht University, the Netherlands
10. Institute of Cardiovascular Science, Faculty of Population Health Sciences, University College London, London, UK

**Word count**

Abstract: 252 words; Text, excluding references, acknowledgements and figure legends: 3117 words.

**Correspondence**

Alicia Uijl (a.uijl@umcutrecht.nl)

Julius Center for Health Sciences and Primary Care, University Medical Center Utrecht

Heidelberglaan 100, 3508 GA Utrecht, the Netherlands



For peer review only - http://bmjopen.bmj.com/site/about/guidelines.xhtml

**Abstract**

*Objective:* We examined temporal heart failure (HF) prescription patterns in a large representative sample of real-world patients in the UK, using electronic health records (EHR).

*Methods:* From primary and secondary care EHR, we identified 85,732 patients with a HF diagnosis between 2002-2015. Almost 50% of HF patients were women and the median age was 79.1 [interquartile range 70.2-85.7] years, with age at diagnosis increasing over time.

*Results:* We found several trends in pharmacological HF management, including increased beta-blocker prescriptions over time (29% in 2002-2005 and 54% in 2013-2015), which was not observed for mineralocorticoid receptor-antagonists (MR-antagonists) (18% in 2002-2005 and 18% in 2013-2015); higher prescription rates of loop diuretics in women and elderly patients together with lower prescription rates of angiotensin converting enzyme-inhibitors and/or angiotensin II receptor blockers , beta-blockers, or MR-antagonists in these patients; and little change in medication prescription rates occurred after 6 months of HF diagnosis; and lastly, patients hospitalised for HF who had no follow-up in primary care had considerably lower prescription rates compared to patients with a HF diagnosis in primary care with or without HF hospitalisation.

*Conclusion:* In the general population, the use of MR-antagonists for HF remained low and did not change throughout 13 years of follow up. With large differences between HF patients, with lowest prescription rates observed in women and elderly patients, and those not followed-up in primary care, these findings suggest HF management can be improved by focusing effort and healthcare resources towards these subgroup and communication between primary and secondary care.

**Keywords:** Prescription patterns, ACE-inhibitors, ARB, RAS-inhibitors, Beta-blockers, MRA, Loop diuretics, Heart failure, Electronic health records.



For peer review only - http://bmjopen.bmj.com/site/about/guidelines.xhtml

**Article summary**

Strengths and limitations of the study

- Large cohort of HF patients from primary and secondary care
- Long follow-up period of almost 15 years
- Unable to differentiate between HF subphenotypes such as HF with reduced, mid-range or preserved ejection fraction.
- Unknown treatment eligibility, contraindications or intolerances that may affect the choice of treatment

For peer review only



For peer review only - http://bmjopen.bmj.com/site/about/guidelines.xhtml

**Abbreviations**

| | |
|---|---|
| HF | Heart failure |
| EHR | Electronic health records |
| CPRD | Clinical Practice Research Datalink |
| HES | Hospital Episode Statistics |
| ONS | Office for National Statistics |
| COPD | Chronic obstructive pulmonary disease |
| RAS-inhibitors | Angiotensin converting enzyme-inhibitors and/or angiotensin II receptor blockers |
| MR-antagonists | Mineralocorticoid receptor-antagonists |
| HFrEF | Heart failure with reduced ejection fraction |
| HFpEF | Heart failure with preserved ejection fraction |

For peer review only



For peer review only - http://bmjopen.bmj.com/site/about/guidelines.xhtml

## Introduction

Heart failure (HF) is a common public health burden, with the prevalence of HF estimated at approximately 500.000 patients in the UK.(1, 2) Once diagnosed, initiation and up titration of guideline recommended therapies can reduce morbidity and mortality, although 5-year survival still remains 20% to 50%.(3, 4)

Several observational studies have assessed treatment uptake in HF patients following their diagnosis. These studies suggest that many patients did not receive guideline recommended therapies, or at low doses with sparse attempts for up titration.(5–8) Optimal treatment for effective disease management seems to be particularly challenging in elderly patients, women or patients with multiple comorbidities and contraindications for treatments.(7, 8) At present, few data are available for prescription trends in HF patients in the general population and even fewer data are available that shed light on medication use in HF patients in the years prior to their HF diagnosis.

The CALIBER resource curates primary and secondary care EHR of 5 million individuals in the UK, including HF diagnosis and medication prescriptions.(9) Given the amount of information available, medication use of all HF patients in the community may be investigated – including those which are underrepresented in randomised clinical trials.

Therefore, we sought to examine HF treatment prescription patterns following a HF diagnosis for the overall population as well as specific subgroups based on gender (e.g. women), age (e.g. elderly), social economic status and healthcare setting (e.g. primary care or secondary care), in a large representative sample of real-world patients in the UK, using electronic health records (EHR). (10)

For peer review only



For peer review only - http://bmjopen.bmj.com/site/about/guidelines.xhtml

## Methods

*Data source*

Patients were selected from linked EHR in the UK, which consist of three linked databases: The Clinical Practice Research Datalink (CPRD) with primary care EHR, Hospital Episodes Statistics (HES) containing coded diagnoses and surgical procedures from inpatient hospital admissions, and the Office for National Statistics (ONS) registry containing cause-specific mortality data.(10) Previous work has shown that these patients are representative of the general population in the UK.(11, 12)

*Study population*

Patients were included at their first record of HF from CPRD or HES between January 1st 2002 and December 31st 2015. In CPRD, events were defined by a diagnosis of HF based on Read (version 2) controlled clinical terminology codes (NHS coded clinical terms) and in HES by a diagnosis of HF based on ICD-10 codes. The same HF diagnosis codes were used as in previous papers, with in addition several newer Read codes listed in **Table S1**.(4, 13) All patients were eligible for inclusion if they were aged 18 years or older, were registered with a GP for at least one year prior to diagnosis of HF, in a practice that had at least one year of up-to-standard data recording in CPRD (data quality check). The first record of HF from CPRD or HES was considered the index date. Individuals were censored at the earliest date from the date of de-registration in CPRD, the last data collection date of a practice in CPRD, the date of death or at the study end date (31st December 2015). Data on EHR phenotyping variables from HF patients up to 3 years prior to index date were included in this study.



For peer review only - http://bmjopen.bmj.com/site/about/guidelines.xhtml

*Patient and public involvement*

There was no patient or public involvement in this research.

*EHR phenotyping variables*

Baseline patient characteristics were based on records from CPRD and/or HES prior to index date, including demographics [age, sex, ethnicity, social deprivation] cardiovascular risk factors [smoking, BMI, diastolic blood pressure and systolic blood pressure and estimated glomerular filtration rate], comorbidities [a medical history of atrial fibrillation, chronic obstructive pulmonary disease (COPD), diabetes, hypertension, ischaemic heart disease, valvular disease and history of cancer] and medication prescription.

CPRD includes all prescriptions from the general practice. Prescriptions in CPRD were classified as: RAS-inhibitors (Angiotensin converting enzyme-inhibitors and/or angiotensin II receptor blockers), beta-blockers, mineralocorticoid receptor-antagonists (MR-antagonists) and loop diuretics. Definitions of these variables could be found online at http://www.caliberresearch.org/portal/.

Medication prescription for RAS-inhibitors, beta-blockers, MR-antagonists and loop diuretics was identified between three years prior to HF diagnosis up to three years after HF diagnosis per the following increments: -36 months to -24 months, -24 months to -18 months, -18 months to -12 months, -12 months to -6 months, -6 months to -3 months, -3 months to HF diagnosis, HF diagnosis to +3 months, +3 months to +6 months, +6 months to +12 months, +12 months to +18 months, +18 months to +24 months and +24 to +36 months.

Healthcare setting was characterised as primary care only (no HF hospitalisation), secondary care only (no Read HF diagnosis recorded in primary care) or HF diagnosis in both primary and secondary care. Ethnicity records from CPRD and HES were combined and



For peer review only - http://bmjopen.bmj.com/site/about/guidelines.xhtml

categorised as Caucasian, Asian, Black or Other. Social deprivation was measured as quintiles of the index of multiple deprivation of the geographical area of the primary care practice, a score calculated based on seven indices of deprivation: income, employment, health and disability, education, barrier to housing and services, crime and living environment.(14) Smoking status in CPRD was classified as never, ex- or current smokers.

*Statistical analysis*

Patient characteristics were summarised as mean (SD) or median [IQR] for continuous variables and percentages for categorical variables. The percentage of HF patients prescribed pharmacological treatments was calculated per increment and per time period as defined by publication year of previous ESC guidelines (2001, 2005, 2008 and 2012)(1, 15–18): 2002-2005, 2006-2008, 2009-2012 and 2013-2015. In addition to the overall cohort, we investigated several subgroups: age (< vs. ≥ 75 years old), sex (men vs. women), social economic status (lowest quintile of social deprivation vs. the rest) and setting (only follow-up in primary care vs. only in secondary care vs. follow-up in primary care after HF hospitalisation). All analyses were performed using R version 3.6.1.

## Results

*Baseline characteristics*

We identified 85,732 patients with a HF diagnosis. The study flow diagram could be found in **Figure S1**. Median follow-up after HF diagnosis (index date) was 2.1 years [0.6 – 4.5] years. **Table 1** shows the overall baseline patient characteristics and per time period 2002-2005, 2006-2008, 2009-2012 and 2013-2015. Almost 50% of patients were women and the median age was 79.1 [70.2 - 85.7] years, with age at HF diagnosis increasing over time. Overall, many



For peer review only - http://bmjopen.bmj.com/site/about/guidelines.xhtml

For peer review only

HF patients had comorbidities, most common were hypertension (61%), ischaemic heart disease (44%) and atrial fibrillation (37%), with increasing numbers of patients with comorbidities over time. Approximately 40% (n= 34,489) of patients were followed-up in primary care after a HF hospitalisation, 20% (n= 15,330) of patients were only known in primary care and never hospitalised for HF and the remaining 40% (n= 35,913) of patients had no follow-up in primary care after HF hospitalisation.

*Overall prescription patterns*

Overall prescription patterns are shown in **Figure 1**. Many patients were prescribed medication before HF diagnosis, especially RAS-inhibitors (20% in 2002-2005 to 46% in 2013-2015). Over time, beta-blocker prescription after HF diagnosis increased from 30% in 2002-2005 to 55% in 2013-2015. Throughout the follow up of 13 years, there were little observed changes for MR-antagonist uptake, this remained at 20% throughout time after HF diagnosis. The largest observed changes in prescription patterns occurred between 6 months before and after HF diagnosis (**Figure 1**). Approximately 20% of HF patients were prescribed a loop diuretic up to three years prior to HF diagnosis.

*Setting-specific prescription patterns*

Setting-specific prescription patterns are shown in **Figure 2**. Patients followed-up in primary care after HF hospitalisation had the highest prescription rates for all types of medication. Over time, the prescription for loop-diuretics, RAS-inhibitors and beta-blockers converged together. In these patients the prescription for MR-antagonists increased over time after HF diagnosis from 20% in 2002-2005 to 30% in 2013-2015.



For peer review only - http://bmjopen.bmj.com/site/about/guidelines.xhtml

Patients known in primary care but never hospitalised for HF had lower prescription rates for all types of treatment compared to patients with primary care follow-up and at least one HF hospitalisation. Mainly loop diuretics were less prescribed in these patients and the prescription of loop diuretics decreased over time with 65% of patients receiving loop diuretics after HF diagnosis in 2002-2005 compared to just over 40% in 2013-2015.

Patients hospitalised for HF but without a HF diagnosis in primary care, had the lowest prescriptions rates for loop diuretics, RAS-inhibitors and beta-blockers, which remained stable over time (50%, 45%, and 45% in 2013-2015 respectively). MR-antagonists were only prescribed in 13% of patients after HF diagnosis, this was similar for each time period.

*Age-specific prescription patterns*

Differences in prescription according to age categories are shown in **Figure 3**. The observed increase in prescriptions for RAS-inhibitors, beta-blockers, and MR-antagonists between 6 months before HF diagnosis to 6 months after HF diagnosis was less pronounced in elderly patients. The average increase in elderly patients was 12%, 7%, 8% for RAS-inhibitors, beta-blockers and MR-antagonists respectively, while younger patients had an average increase of 23%, 19% and 13% for RAS-inhibitors, beta-blockers and MR-antagonists respectively. On the other hand, a higher proportion of elderly patients were treated with loop-diuretics compared to younger patients, both before and after HF diagnosis (45% before and 63% after HF diagnosis in elderly compared to 27% before and 47% after HF diagnosis for younger patients in 2013-2015). After HF diagnosis, a higher percentage of younger patients were prescribed with RAS-inhibitors and beta-blockers compared to older patients.



For peer review only - http://bmjopen.bmj.com/site/about/guidelines.xhtml

*Sex-specific prescription patterns*

Differences in prescription between men and women are shown in **Figure 4**. Loop diuretics were prescribed in a higher proportion of women compared to men, this difference was already present prior to HF diagnosis where 6 months before diagnosis 30% of women and 20% of men were prescribed a loop diuretic. After HF diagnosis, the most prescribed medication for women was a loop diuretic, while a higher proportion of men were prescribed a RAS-inhibitor. Men were also more often prescribed RAS-inhibitors, beta-blockers and MR-antagonists after HF diagnosis compared to women.

*Social economic status-specific prescription patterns*

Social economic status-specific prescription patterns are shown in **Figure 5**. We did not observe any discernible differences between patients in low vs. high social-economic areas (highest quintile of social economic deprivation).

## Discussion

In this large-scale study of 85,732 HF patients we investigated treatment prescription patterns in a representative sample of real-world patients with HF in the UK between 2002 and 2015. We found three important trends in pharmacological HF management: a) increased use of beta-blockers, whereas there was no increased uptake of MR-antagonists over 13 years follow up; b) prescription rates remained almost unchanged after the first 6 months following a HF diagnosis; and lastly, c) higher rates of loop diuretics in women and elderly patients together with lower prescription rates for RAS-inhibitors, beta-blockers, or MR-antagonists.

For peer review only

*Temporal trends in heart failure medication*

Even though prescription rates increased over time from 2002 to 2015, overall prescription rates remained low. This is in line with previously published studies.(5–8, 19) Low prescription rates could be attributed to the mixed HF cases found in EHR. We were unable to distinguish HF with reduced ejection fraction (HFrEF), HF with mid-range ejection fraction, and HF with preserved ejection fraction (HFpEF) based on medical records, thereby including known differences in treatment recommendations for these HF phenotypes.(1)

We found no major differences in prescription behaviour after the publication of ESC guidelines, however we did observe the gradual increase of beta-blockers as one of the cornerstones of HF treatment. RAS-inhibitors were prescribed in a high proportion of patients throughout the years of the study, presumably because the first clinical trials in HFrEF showing a beneficial effect were from the late 1980s and early 1990s.(20) Surprisingly, we found lower than expected prescription rates for MR-antagonists, which persisted over the years included in this study. This is in spite of multiple clinical trials which have shown benefit in HFrEF patients.(21) Besides HFrEF trials, a post-hoc analysis of the TOPCAT trial in 2015 (Spironolactone, a MR-antagonist, for HFpEF) reported regional differences between Americas and Russia/Georgia, where the American patients showed clinical benefits.(22) The American College of Cardiology/American Heart Association focused update on HF management in 2017 gave spironolactone a grade IIb recommendation, thereby stimulating that selected HFpEF patients could be treated with spironolactone to decrease re-hospitalisations.(23)

*Heart failure medication initiation following diagnosis*

Most activity in treatment prescription behaviour was observed between 6 months before to 6 months after HF diagnosis. After the 6 month mark we did not observe many patients starting



For peer review only - http://bmjopen.bmj.com/site/about/guidelines.xhtml

any of the medication investigated. This is in line with previous studies showing that there are few changes in medication use and little up titration of medication after treatment initiation.(5, 24) This leaves room for improvement in starting treatment at any time point following a HF diagnosis, for example if patients hospitalised with acute HF do not immediately tolerate negative inotropic medication such as beta-blockers.

*Impact of heart failure hospitalisation on medication prescription*

We found differences in prescription patterns between patients with a HF diagnosis recorded in different settings. Patients with a primary care HF diagnosis without HF hospitalisation had much lower prescription rates of loop diuretics compared to patients with a HF diagnosis recorded in both primary and secondary care. It could be that these patients have less severe fluid overload or symptoms that requires alleviation by loop diuretics, and thus less severe HF.

Previously it was shown that there are differences in overall five-year survival of patients with HF diagnosis recorded in primary care only, secondary care only and in both, with the worst survival seen in HF patients identified only in secondary care and the best survival for HF patients identified in primary care with or without hospitalisation for HF.(4) Here, we advance current knowledge by showing that there are longitudinal differences in HF care of patients with diagnosis recorded in different settings.

In this study almost 40% of patients did not have a GP record of a HF diagnosis after a HF hospitalisation. One reason could be that GPs do not formally register HF with a Read diagnosis code, but rather in free text descriptions. However, there could also be a potential quality of care gap or failure of communication between secondary and primary care, where patients are not treated optimally. Primary care is the basis of many healthcare systems, including the UK. If there is no HF diagnosis recorded in primary care after HF hospitalisation,



For peer review only - http://bmjopen.bmj.com/site/about/guidelines.xhtml

For peer review only

which is shown to indicative for worse survival, rehospitalisation and severity of disease, this could be detrimental for patients.

*Heart failure treatment in women and elderly*

Over time, we observed that HF was diagnosed at a later age, with the median almost 80 years old between 2013-2015. This is also seen in many other developed countries where the mean age of HF diagnosis is over 70 years old.(25, 26)

We observed lower prescription rates in elderly patients compared to younger patients for RAS-inhibitors, beta-blockers and MR-antagonists, although the difference in MR-antagonists was less pronounced. Many elderly patients were already using RAS-inhibitors prior to HF diagnosis, therefore the increase in prescription rate is not as steep as compared to younger HF patients who are prescribed less medication prior to HF diagnosis. This could be explained by the presence of comorbidities, such as atrial fibrillation or hypertension, which are much more prevalent among elderly compared to younger patients, and for which these elderly patients could be prescribed RAS-inhibitors.

Remarkably, the difference between prescription of RAS-inhibitors and beta-blockers prior to HF diagnosis was less than 5% for men and women, and only after the diagnosis of HF was a higher proportion of men prescribed a RAS-inhibitor or beta-blocker. This could potentially be related to the fact that elderly women are more likely to develop HFpEF and therefore tend to be treated symptomatically with loop diuretics, rather than with RAS-inhibitors and beta-blockers. However, the literature also shows that there are differences in treatment prescription in men and women with HFrEF, for which there is no obvious explanation.(27)



For peer review only - http://bmjopen.bmj.com/site/about/guidelines.xhtml

For peer review only

Both elderly patients and women received more loop diuretics. However, this could potentially be harmful, especially for elderly, since loop diuretics could lead to electrolyte disturbances and acute kidney injury.(28) Elderly patients are often excluded or underrepresented in clinical trials, therefore current recommendations lack convincing evidence in the elderly population. However, recently a large meta-analysis reported a significant effect of beta-blockers on overall mortality regardless of age.(29) These studies indicate that elderly patients also benefit from HF-specific medication and should be a choice of treatment for these patients, besides loop diuretics for symptom alleviation. However, elderly patients might have more contraindications or intolerances to RAS-inhibitors, beta-blockers and MR-antagonists and might therefore be more often treated with loop diuretics for symptom control.

*Strengths and limitations*

Strengths of this study are the large cohort of HF patients and a long follow-up period. Patient records available are representative of the general UK population, which provides evidence for the validity of using these EHR for research.(11, 12) However, we were limited by the inability to differentiate between HF phenotypes based on medical records, since there was no access to detailed echocardiography estimates to assess systolic function. Nor did we have information on NYHA class or NT-proBNP biomarker levels. Furthermore, we only had medication prescription available in primary care, not in hospital care. However, CPRD includes all prescriptions from community. Treatments administered during a hospital admission or discharge were not reported, such as intravenous inotropic agents. We were also unable to assess patients' symptom class (which would affect their eligibility for treatments such as MRA-antagonists), and contraindications or intolerances that may affect the choice of medication.



For peer review only - http://bmjopen.bmj.com/site/about/guidelines.xhtml

*Conclusion*

The results of this population-based study of over 80,000 patients with heart failure in England shows variable increases in uptake of evidence-based treatments, with no change in prescription of MR-antagonists over 13 years, but an increase in beta-blocker use. Large differences were observed between HF patient groups, with lowest prescription rates of RAS-inhibitors, beta-blockers and MR-antagonists in women, elderly patients, and those without a HF diagnosis in primary care. These findings suggest HF management can be improved by focusing effort and healthcare resources on improving communication between primary and secondary care. There is still a need for effective implementation of guideline-recommended therapies in real-world HF care.

For peer review only



For peer review only - http://bmjopen.bmj.com/site/about/guidelines.xhtml

**Acknowledgements**

This study was approved by the Medicines and Healthcare Products Regulatory Agency (UK) Independent Scientific Advisory Committee protocol reference: 17_015, under Section 251 (NHS Social Care Act 2006). This study is based in part on data from the Clinical Practice Research Datalink obtained under license from the UK Medicines and Healthcare products Regulatory Agency. The data are provided by patients and collected by the NHS as part of their care and support. The interpretation and conclusions contained in this study are those of the author(s) alone. Hospital Episode Statistics Copyright (2019) is reused with the permission of The Health & Social Care Information Centre. All rights reserved. "This study was carried out as part of the CALIBER © resource (https://www.ucl.ac.uk/health-informatics/caliber and https://www.caliberresearch.org/). CALIBER, led from the UCL Institute of Health Informatics, is a research resource providing validated electronic health record phenotyping algorithms and tools for national structured data sources.

**a. Contributor statement**

AU has designed the research, analysed and interpreted the data and drafted the manuscript. IV, AH, FA and SK have designed the research, interpreted the data, critically revised the manuscript and supervised AU. SD, HH, AS, JC and DG have interpreted the data and critically revised the manuscript.

**b. Competing interests**

The authors report no conflict of interest.



For peer review only - http://bmjopen.bmj.com/site/about/guidelines.xhtml

**c. Funding**

This study is part of the BigData@Heart program that has received funding from the Innovative Medicines Initiative 2 Joint Undertaking under grant agreement No 116074. This Joint Undertaking receives support from the European Union's Horizon 2020 research and innovation programme and EFPIA.

This work was supported by Health Data Research UK [grant number N/A], which receives its funding from Health Data Research UK Ltd (NIWA1) funded by the UK Medical Research Council, Engineering and Physical Sciences Research Council, Economic and Social Research Council, Department of Health and Social Care (England), Chief Scientist Office of the Scottish Government Health and Social Care Directorates, Health and Social Care Research and Development Division (Welsh Government), Public Health Agency (Northern Ireland), British Heart Foundation, and the Wellcome Trust. Work at the University College London Institute of Health Informatics and Institute of Cardiovascular Science is supported by a British Heart Foundation Accelerator Award (AA/18/6/24223).

**FA** is supported by UCL Hospitals NIHR Biomedical Research Centre. **IV** is supported by a grant from the Dutch Heart Foundation [grant DHF project 'Facts and Figures']. **SD** is supported by an Alan Turing Fellowship. **HH** is supported by an NIHR Senior Investigator Award. **ADS** is funded by a post-doctoral fellowship from THIS Institute. **JC** received research grants from Bayer, Novartis and Vifor and honoraria for steering committees from Amgen, Bayer, Novartis and Servier.



For peer review only - http://bmjopen.bmj.com/site/about/guidelines.xhtml

### d. Data sharing statement

All data were provided anonymised and are not publicly available due to their sensitive nature. Data may be obtained from the Clinical Practice Research Datalink (https://www.cprd.com). EHR phenotypes are available from the CALIBER resource (https://www.caliberresearch.org). The protocol may be obtained via the Clinical Practice Research Datalink under protocol reference: 17_015. No additional data is available.

For peer review only

## References

1. Ponikowski P, Voors AA, Anker SD, et al. 2016 ESC Guidelines for the diagnosis and treatment of acute and chronic heart failure: The Task Force for the diagnosis and treatment of acute and chronic heart failure of the European Society of Cardiology (ESC). Developed with the special contribution . Eur. J. Heart Fail. 2016;18:891–975.

2. British Heart Foundation Centre on Population Approaches for Non-Communicable Disease Prevention. British Heart Foundation Cardiovascular Disease Statistics 2015. 2015.

3. Bleumink GS, Knetsch AM, Sturkenboom MCJM, et al. Quantifying the heart failure epidemic: prevalence, incidence rate, lifetime risk and prognosis of heart failure The Rotterdam Study. Eur. Heart J. 2004;25:1614–1619.

4. Koudstaal S, Pujades-Rodriguez M, Denaxas S, et al. Prognostic burden of heart failure recorded in primary care, acute hospital admissions, or both: a population-based linked electronic health record cohort study in 2.1 million people. Eur. J. Heart Fail. 2017;19:1119-1127.

5. Deschaseaux C, McSharry M, Hudson E, Agrawal R, Turner SJ. Treatment Initiation Patterns, Modifications, and Medication Adherence Among Newly Diagnosed Heart Failure Patients: A Retrospective Claims Database Analysis. J. Manag. care Spec. Pharm. 2016;22:561–571.

6. Bress AP, King JB, Brixner D, et al. Pharmacotherapy Treatment Patterns, Outcomes, and Health Resource Utilization Among Patients with Heart Failure with Reduced Ejection Fraction at a U.S. Academic Medical Center. Pharmacotherapy 2016;36:174–186.

7. Stork S, Handrock R, Jacob J, et al. Treatment of chronic heart failure in Germany: a retrospective database study. Clin. Res. Cardiol. 2017;106:923-932.

8. Chin KL, Skiba M, Tonkin A, et al. The treatment gap in patients with chronic systolic heart failure: a systematic review of evidence-based prescribing in practice. Heart Fail. Rev.



For peer review only - http://bmjopen.bmj.com/site/about/guidelines.xhtml

2016;21:675–697.

9. Denaxas S, Gonzalez-Izquierdo A, Direk K, et al. UK phenomics platform for developing and validating electronic health record phenotypes: CALIBER. J. Am. Med. Inform. Assoc. 2019;26:1545-1559.

10. Denaxas SC, George J, Herrett E, et al. Data resource profile: cardiovascular disease research using linked bespoke studies and electronic health records (CALIBER). Int. J. Epidemiol. 2012;41:1625–1638.

11. Herrett E, Thomas SL, Schoonen WM, Smeeth L, Hall AJ. Validation and validity of diagnoses in the General Practice Research Database: a systematic review. Br. J. Clin. Pharmacol. 2010;69:4–14.

12. Burns EM, Rigby E, Mamidanna R, et al. Systematic review of discharge coding accuracy. J. Public Health. 2012;34:138–148.

13. Uijl A, Koudstaal S, Direk K, et al. Risk factors for incident heart failure in age- and sex-specific strata: a population-based cohort using linked electronic health records. Eur. J. Heart Fail. 2019;21:1197-1206.

14. McLennan D, Barnes H, Noble M, Davies J, Garratt E. The English indices of deprivation, 2010: Technical Report. London: HMSO. 2011.

15. Remme WJ, Swedberg K. Guidelines for the diagnosis and treatment of chronic heart failure. Eur. Heart J. 2001;22:1527–1560.

16. Swedberg K, Drexler H, Follath F, et al. Guidelines for the diagnosis and treatment of chronic heart failure: executive summary ( update 2005 ) The Task Force for the Diagnosis and Treatment of Chronic Heart Failure of the European Society of Cardiology. Eur. Heart J. 2005:1115–1140.

17. Dickstein K, Cohen-Solal A, Filippatos G, et al. ESC guidelines for the diagnosis and



For peer review only - http://bmjopen.bmj.com/site/about/guidelines.xhtml

treatment of acute and chronic heart failure 2008: the Task Force for the diagnosis and treatment of acute and chronic heart failure 2008 of the European Society of Cardiology. Developed in collaboration with the Heart. Eur. J. Heart Fail. 2008;10:933–989.

18. McMurray JJV, Adamopoulos S, Anker SD, et al. ESC Guidelines for the diagnosis and treatment of acute and chronic heart failure 2012: The Task Force for the Diagnosis and Treatment of Acute and Chronic Heart Failure 2012 of the European Society of Cardiology. Developed in collaboration with the Heart Failure Association (HFA) of the ESC. Eur. Heart J. 2012;33:1787–1847.

19. Peters-Klimm F, Muller-Tasch T, Schellberg D, et al. Guideline adherence for pharmacotherapy of chronic systolic heart failure in general practice: a closer look on evidence-based therapy. Clin. Res. Cardiol. 2008;97:244–252.

20. SOLVD Investigators, Yusuf S, Pitt B, Davis CE, Hood WB, Cohn JN. Effect of enalapril on survival in patients with reduced left ventricular ejection fractions and congestive heart failure. N. Engl. J. Med. 1991;325:293–302.

21. Pitt B, Zannad F, Remme WJ, et al. The Effect of Spironolactone on Morbidity and Mortality in Patients with Severe Heart Failure. N. Engl. J. Med. 1999;341:709–717.

22. Pfeffer MA, Claggett B, Assmann SF, et al. Regional Variation in Patients and Outcomes in the Treatment of Preserved Cardiac Function Heart Failure With an Aldosterone Antagonist (TOPCAT) Trial. Circulation 2015;131:34–42.

23. Yancy CW, Jessup M, Bozkurt B, et al. 2017 ACC/AHA/HFSA Focused Update of the 2013 ACCF/AHA Guideline for the Management of Heart Failure: A Report of the American College of Cardiology/American Heart Association Task Force on Clinical Practice Guidelines and the Heart Failure Society of Amer. J. Am. Coll. Cardiol. 2017;68:1476–1488.

24. Greene SJ, Fonarow GC, DeVore AD, et al. Titration of Medical Therapy for Heart Failure



For peer review only - http://bmjopen.bmj.com/site/about/guidelines.xhtml

With Reduced Ejection Fraction. J. Am. Coll. Cardiol. 2019;73:2365–2383.

25. Brugts JJ, Linssen GCM, Hoes AW, Brunner-La Rocca HP. Real-world heart failure management in 10,910 patients with chronic heart failure in the Netherlands. Netherlands Hear. J. 2018;26:272–279.

26. Savarese G, Vasko P, Jonsson Å, Edner M, Dahlström U, Lund LH. The Swedish Heart Failure Registry: a living, ongoing quality assurance and research in heart failure. Ups. J. Med. Sci. 2019;124:65–69.

27. Dewan P, Rørth R, Jhund PS, et al. Differential Impact of Heart Failure With Reduced Ejection Fraction on Men and Women. J. Am. Coll. Cardiol. 2019;73:29–40.

28. Täger T, Fröhlich H, Seiz M, Katus HA, Frankenstein L. READY: relative efficacy of loop diuretics in patients with chronic systolic heart failure—a systematic review and network meta-analysis of randomised trials. Heart Fail. Rev. 2019;24:461-472.

29. Kotecha D, Manzano L, Krum H, et al. Effect of age and sex on efficacy and tolerability of beta blockers in patients with heart failure with reduced ejection fraction: individual patient data meta-analysis. BMJ 2016;353:i1855.

For peer review only



For peer review only - http://bmjopen.bmj.com/site/about/guidelines.xhtml

## Figure legends

**Central illustration Figure 1**

**Legend Figure 1.** The percentage of HF patients receiving prescriptions of RAS-inhibitors, beta-blockers, MR-antagonists, loop diuretics per months since HF diagnosis. RAS-inhibitors = ACE-inhibitors and/or ARB; MR-antagonists = mineralocorticoid receptor antagonists.

**Legend Figure 2.** The percentage of HF patients receiving prescriptions of RAS-inhibitors, beta-blockers and MRAs per months since HF diagnosis, stratified by setting (primary care only, secondary care only, both primary and secondary care). RAS-inhibitors = ACE-inhibitors and/or ARB; MR-antagonists = mineralocorticoid receptor antagonists.

**Legend Figure 3.** The percentage of HF patients receiving prescriptions of RAS-inhibitors, beta-blockers and MRAs per months since HF diagnosis, stratified by age. RAS-inhibitors = ACE-inhibitors and/or ARB; MR-antagonists = mineralocorticoid receptor antagonists.

**Legend Figure 4.** The percentage of HF patients receiving prescriptions of RAS-inhibitors, beta-blockers and MRAs per months since HF diagnosis, stratified by sex. RAS-inhibitors = ACE-inhibitors and/or ARB; MR-antagonists = mineralocorticoid receptor antagonists.

**Legend Figure 5.** The percentage of HF patients receiving prescriptions of RAS-inhibitors, beta-blockers and MRAs per months since HF diagnosis, stratified by social status (highest quintile of social deprivation vs. the rest). RAS-inhibitors = ACE-inhibitors and/or ARB; MR-antagonists = mineralocorticoid receptor antagonists.

For peer review only

**Table 1. Patients characteristics of heart failure patients between 2002 and 2015**

| | Overall | 2002 - 2005 | 2006 - 2008 | 2009 - 2012 | 2013 - 2015 | % missing |
|---|---|---|---|---|---|---|
| n | 85732 | 25366 | 17715 | 26114 | 16537 | |
| **Demographics** | | | | | | |
| Age (Years, median [IQR]) | 79.1 [70.2, 85.7] | 78.7 [70.7, 84.9] | 78.7 [ 69.9, 85.4] | 79.5 [70.1, 86.3] | 79.7 [70.0, 86.4] | 0 |
| Sex (% Women) | 48.6 | 49.3 | 48.4 | 48.4 | 48.0 | 0 |
| Ethnicity (% Caucasian) | 96.5 | 97.5 | 96.9 | 96.1 | 95.1 | 3.5 |
| Social deprivation (% lowest quintile) | 24.3 | 25.1 | 25.0 | 24.0 | 22.9 | 0 |
| **Clinical and lifestyle measurements** | | | | | | |
| SBP (mmHg, mean (sd)) | 136.2 (20.7) | 140.6 (22.3) | 135.9 (20.7) | 134.6 (20.0) | 132.9 (18.7) | 13.0 |
| DBP (mmHg, mean (sd)) | 76.2 (12.0) | 78.4 (12.0) | 76.2 (12.0) | 75.4 (12.0) | 74.4 (11.6) | 13.0 |
| BMI (kg/$m^2$, mean (sd)) | 28.6 (6.6) | 28.2 (6.4) | 28.4 (6.6) | 28.7 (6.8) | 28.8 (6.8) | 54.0 |
| eGFR (min/m2/1.73mL, median [IQR]) | 58.4 [ 45.3, 72.1] | 54.7 [43.4, 66.1] | 56.5 [44.3, 68.8] | 60.5 [46.3, 75.3] | 62.9 [47.5, 78.2] | 24.0 |
| Smoking status (% Current) | 20.8 | 22.3 | 20.0 | 20.4 | 20.5 | 38.7 |
| **Medical history (%)**[¶] | | | | | | |
| Atrial Fibrillation | 36.6 | 28.4 | 36.3 | 40.6 | 43.0 | - |
| COPD | 17.9 | 14.8 | 17.3 | 19.5 | 21.0 | - |
| Diabetes | 22.3 | 18.1 | 22.2 | 23.7 | 26.7 | - |
| Hypertension | 60.7 | 46.0 | 60.7 | 67.9 | 72.0 | - |
| Ischaemic heart disease | 44.2 | 39.0 | 46.0 | 46.4 | 46.8 | - |
| Valvular disease | 16.5 | 9.5 | 14.9 | 19.9 | 23.8 | - |
| **Medication prescription up to 3 months after HF diagnosis (%)**[¶] | | | | | | |
| RAS-inhibitors | 60.8 | 59.6 | 63.5 | 62.0 | 57.6 | - |
| Beta-blockers | 42.5 | 28.9 | 41.0 | 49.3 | 54.1 | - |
| MR-antagonists | 18.0 | 18.4 | 17.9 | 17.6 | 18.2 | - |
| Loop diuretics | 63.0 | 68.4 | 63.5 | 61.1 | 57.0 | - |

For peer review only

**Legend Table 1.** * Assessed by index of multiple deprivation, § denotes prior medical history of given comorbidity, ¤ Medication 12 months prior to index date, Mean (SD) = Mean (Standard deviation), Median [IQR] = Median [Interquartile range], CPRD = Clinical Practice Research Datalink, SBP = systolic blood pressure, DBP = Diastolic blood pressure, BMI = Body Mass Index, eGFR = estimated glomerular filtration rate, COPD = Chronic Obstructive Pulmonary Disease, RAS-inhibitors = ACE-inhibitors and/or angiotensin II receptor blockers, MR-antagonists = mineralocorticoid receptor antagonist. [¶]Medical conditions and prescriptions were considered absent if not recorded.

For peer review only



For peer review only - http://bmjopen.bmj.com/site/about/guidelines.xhtml

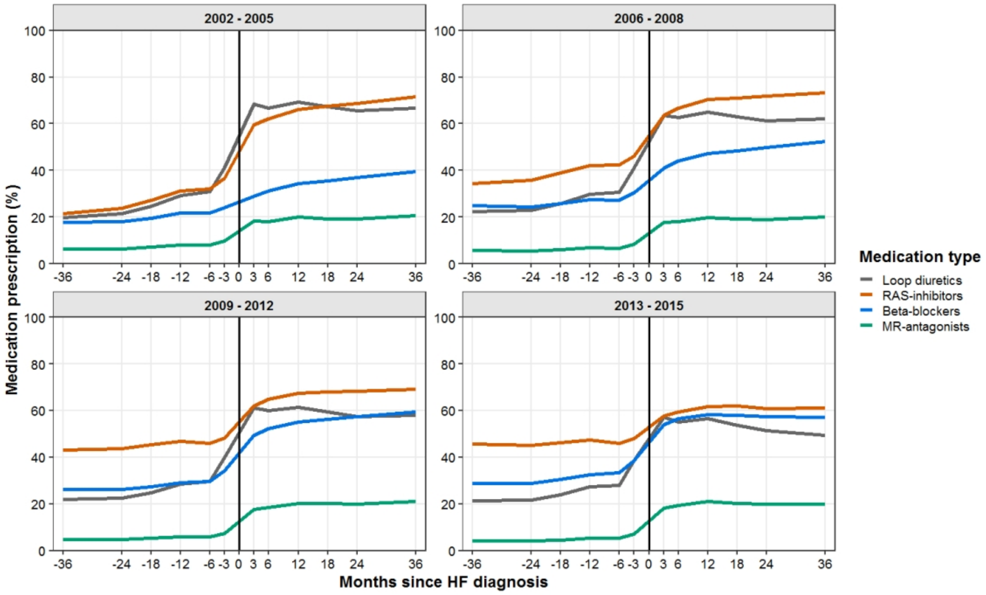


figure 1

127x76mm (300 x 300 DPI)

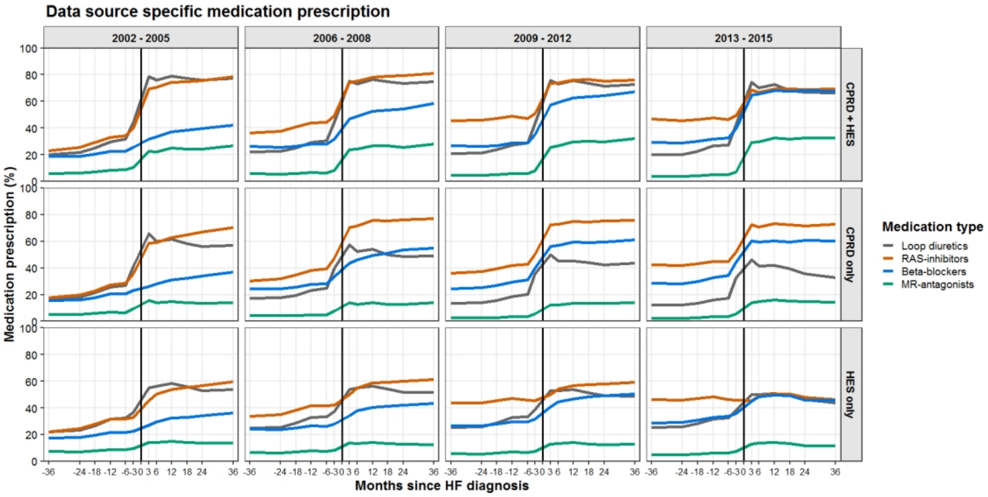


figure 2

152x76mm (300 x 300 DPI)

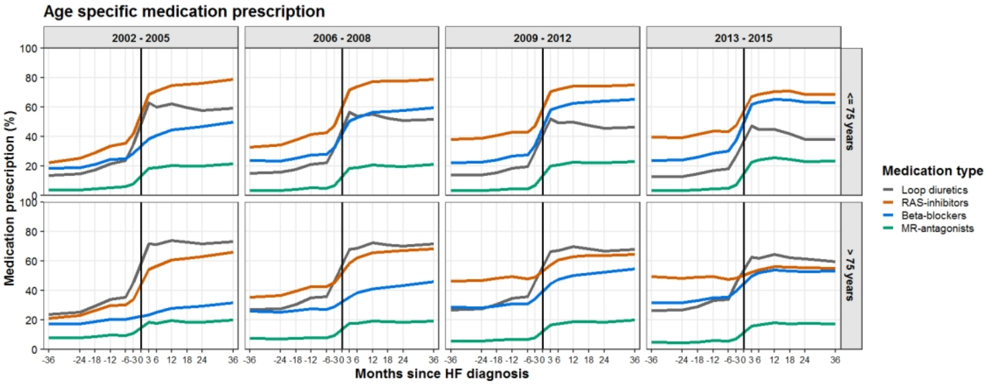


figure 3

152x59mm (300 x 300 DPI)

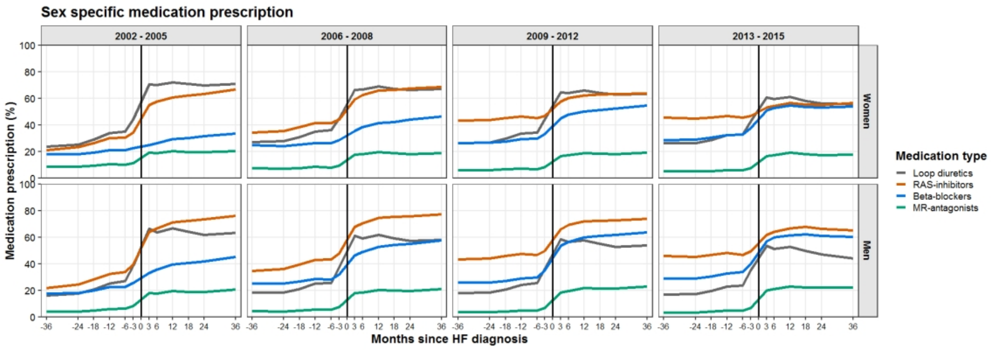


figure 4

169x59mm (300 x 300 DPI)

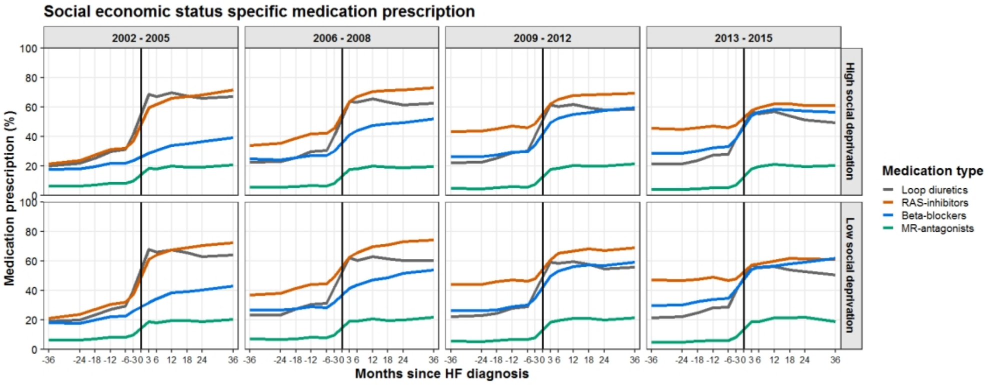


figure 5

152x59mm (300 x 300 DPI)

**Supplemental material**

**Figure S1 – study flow diagram**

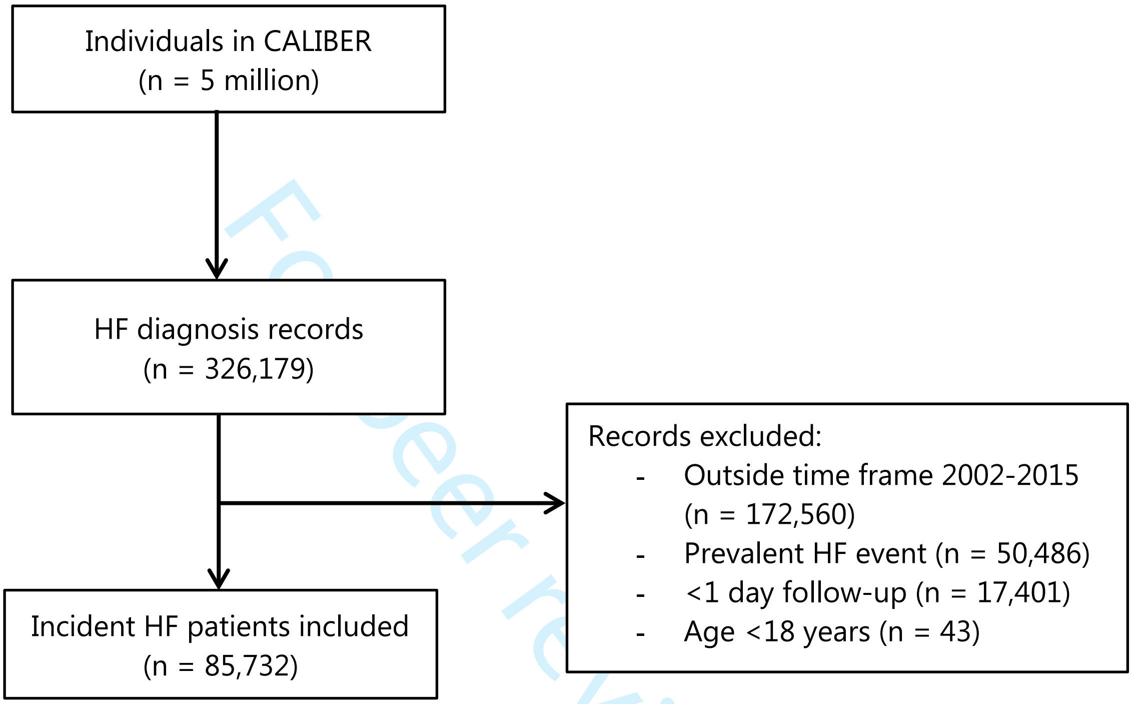


For peer review only

**Table S1 – additional READ codes used to identify heart failure in the Clinical Practice Research Datalink**

| CPRD* Heart Failure READ codes |
|---|
| 585g.00, G5yyC00, G5yyA00, G583.12, G583.11, G583.00, G5yy900, 585f.00 |

**Legend Table S1**. * CPRD = Clinical Practice Research Datalink

For peer review only

STROBE Statement—Checklist of items that should be included in reports of ***cohort studies***

| | Item No | Recommendation | Page No |
|---|---|---|---|
| **Title and abstract** | 1 | (*a*) Indicate the study's design with a commonly used term in the title or the abstract<br>(*b*) Provide in the abstract an informative and balanced summary of what was done and what was found | 1, 3 |
| **Introduction** | | | |
| Background/rationale | 2 | Explain the scientific background and rationale for the investigation being reported | 6 |
| Objectives | 3 | State specific objectives, including any prespecified hypotheses | 6 |
| **Methods** | | | |
| Study design | 4 | Present key elements of study design early in the paper | 7 |
| Setting | 5 | Describe the setting, locations, and relevant dates, including periods of recruitment, exposure, follow-up, and data collection | 7 |
| Participants | 6 | (*a*) Give the eligibility criteria, and the sources and methods of selection of participants. Describe methods of follow-up<br>(*b*) For matched studies, give matching criteria and number of exposed and unexposed | 7 |
| Variables | 7 | Clearly define all outcomes, exposures, predictors, potential confounders, and effect modifiers. Give diagnostic criteria, if applicable | 8 |
| Data sources/ measurement | 8* | For each variable of interest, give sources of data and details of methods of assessment (measurement). Describe comparability of assessment methods if there is more than one group | - |
| Bias | 9 | Describe any efforts to address potential sources of bias | - |
| Study size | 10 | Explain how the study size was arrived at | - |
| Quantitative variables | 11 | Explain how quantitative variables were handled in the analyses. If applicable, describe which groupings were chosen and why | - |
| Statistical methods | 12 | (*a*) Describe all statistical methods, including those used to control for confounding<br>(*b*) Describe any methods used to examine subgroups and interactions<br>(*c*) Explain how missing data were addressed<br>(*d*) If applicable, explain how loss to follow-up was addressed<br>(*e*) Describe any sensitivity analyses | 9 |
| **Results** | | | |
| Participants | 13* | (a) Report numbers of individuals at each stage of study—eg numbers potentially eligible, examined for eligibility, confirmed eligible, included in the study, completing follow-up, and analysed<br>(b) Give reasons for non-participation at each stage<br>(c) Consider use of a flow diagram | 9 |
| Descriptive data | 14* | (a) Give characteristics of study participants (eg demographic, clinical, social) and information on exposures and potential confounders<br>(b) Indicate number of participants with missing data for each variable of interest<br>(c) Summarise follow-up time (eg, average and total amount) | 9 - 10 |
| Outcome data | 15* | Report numbers of outcome events or summary measures over time | 10 - 12 |

For peer review only

| | | | |
|---|---|---|---|
| Main results | 16 | (*a*) Give unadjusted estimates and, if applicable, confounder-adjusted estimates and their precision (eg, 95% confidence interval). Make clear which confounders were adjusted for and why they were included<br>(*b*) Report category boundaries when continuous variables were categorized<br>(*c*) If relevant, consider translating estimates of relative risk into absolute risk for a meaningful time period | 10 - 12 |
| Other analyses | 17 | Report other analyses done—eg analyses of subgroups and interactions, and sensitivity analyses | - |
| **Discussion** | | | |
| Key results | 18 | Summarise key results with reference to study objectives | 12 |
| Limitations | 19 | Discuss limitations of the study, taking into account sources of potential bias or imprecision. Discuss both direction and magnitude of any potential bias | 16 |
| Interpretation | 20 | Give a cautious overall interpretation of results considering objectives, limitations, multiplicity of analyses, results from similar studies, and other relevant evidence | 13 - 15 |
| Generalisability | 21 | Discuss the generalisability (external validity) of the study results | 13 - 15 |
| **Other information** | | | |
| Funding | 22 | Give the source of funding and the role of the funders for the present study and, if applicable, for the original study on which the present article is based | 2 |

*Give information separately for exposed and unexposed groups.

**Note:** An Explanation and Elaboration article discusses each checklist item and gives methodological background and published examples of transparent reporting. The STROBE checklist is best used in conjunction with this article (freely available on the Web sites of PLoS Medicine at http://www.plosmedicine.org/, Annals of Internal Medicine at http://www.annals.org/, and Epidemiology at http://www.epidem.com/). Information on the STROBE Initiative is available at http://www.strobe-statement.org.

For peer review only

BMJ Open

# Temporal Trends in Heart Failure Medication Prescription in a Population-Based Cohort Study

| | |
|---|---|
| Journal: | *BMJ Open* |
| Manuscript ID | bmjopen-2020-043290.R2 |
| Article Type: | Original research |
| Date Submitted by the Author: | 12-Jan-2021 |
| Complete List of Authors: | Uijl, Alicia; UMC Utrecht, Julius Center for Health Sciences and Primary Care<br>Vaartjes, Ilonca; UMC Utrecht, Julius Center for Health Sciences and Primary Care<br>Denaxas, S; University College London, Institute for Health Informatics<br>Hemingway, Harry; University College London, Institute of Health Informatics<br>Shah, Anoop; University College London, Institute of Health Informatics<br>Cleland, J; Imperial College London, National Heart & Lung Institute<br>Grobbee, Diederick; UMC Utrecht, Julius Center for Health Sciences and Primary Care<br>Hoes, Arno; UMC Utrecht, Julius Center for Health Sciences and Primary Care<br>Asselbergs, Folkert; UMC Utrecht, Department of Cardiology; ICIN-Netherlands Heart Institute, Durrer Center for Cardiogenetic Research<br>Koudstaal, Stefan; UMC Utrecht, Department of Cardiology, Division Heart & Lungs |
| <b>Primary Subject Heading</b>: | Epidemiology |
| Secondary Subject Heading: | General practice / Family practice, Cardiovascular medicine, Public health |
| Keywords: | Heart failure < CARDIOLOGY, Cardiac Epidemiology < CARDIOLOGY, PUBLIC HEALTH |
| | |

SCHOLARONE™
Manuscripts

*I, the Submitting Author has the right to grant and does grant on behalf of all authors of the Work (as defined in the below author licence), an exclusive licence and/or a non-exclusive licence for contributions from authors who are: i) UK Crown employees; ii) where BMJ has agreed a CC-BY licence shall apply, and/or iii) in accordance with the terms applicable for US Federal Government officers or employees acting as part of their official duties; on a worldwide, perpetual, irrevocable, royalty-free basis to BMJ Publishing Group Ltd ("BMJ") its licensees and where the relevant Journal is co-owned by BMJ to the co-owners of the Journal, to publish the Work in this journal and any other BMJ products and to exploit all rights, as set out in our licence.*

*The Submitting Author accepts and understands that any supply made under these terms is made by BMJ to the Submitting Author unless you are acting as an employee on behalf of your employer or a postgraduate student of an affiliated institution which is paying any applicable article publishing charge ("APC") for Open Access articles. Where the Submitting Author wishes to make the Work available on an Open Access basis (and intends to pay the relevant APC), the terms of reuse of such Open Access shall be governed by a Creative Commons licence – details of these licences and which Creative Commons licence will apply to this Work are set out in our licence referred to above.*

*Other than as permitted in any relevant BMJ Author's Self Archiving Policies, I confirm this Work has not been accepted for publication elsewhere, is not being considered for publication elsewhere and does not duplicate material already published. I confirm all authors consent to publication of this Work and authorise the granting of this licence.*

For peer review only

**Title:** Temporal Trends in Heart Failure Medication Prescription in a Population-Based Cohort Study

**Authors:** Alicia Uijl PhD[1,2,3,4], Ilonca Vaartjes PhD[1], Spiros Denaxas PhD[3,4,5,6], Harry Hemingway MD PhD[3,4,6], Anoop D. Shah MD PhD[3,4], John Cleland MD PhD[7,8], Diederick E. Grobbee MD PhD[1], Arno W. Hoes MD PhD[1], Folkert W. Asselbergs MD PhD[3,4,9,10], Stefan Koudstaal MD PhD[3,4,9]

**Author affiliations:**

1. Julius Global Health, Julius Center for Health Sciences and Primary Care, University Medical Center Utrecht, Utrecht University, the Netherlands
2. Division of Cardiology, Department of Medicine, Karolinska Institutet, Stockholm, Sweden
3. Institute of Health Informatics, University College London, London, UK
4. Health Data Research UK, London, UK
5. The National Institute for Health Research University College London Hospitals Biomedical Research Centre, University College London, London, UK
6. Alan Turing institute, London, UK
7. Robertson Centre for Biostatistics & Clinical Trials, University of Glasgow, UK
8. National Heart & Lung Institute, Imperial College, London, UK
9. Department of Cardiology, Division Heart & Lungs, University Medical Center Utrecht, Utrecht University, the Netherlands
10. Institute of Cardiovascular Science, Faculty of Population Health Sciences, University College London, London, UK

For peer review only

**Word count**

Abstract: 224 words; Text, excluding references, acknowledgements and figure legends: 2993 words.

**Correspondence**

Alicia Uijl (a.uijl@umcutrecht.nl)

Julius Center for Health Sciences and Primary Care, University Medical Center Utrecht

Heidelberglaan 100, 3508 GA Utrecht, the Netherlands



For peer review only - http://bmjopen.bmj.com/site/about/guidelines.xhtml

**Abstract**

*Objective:* We examined temporal heart failure (HF) prescription patterns in a large representative sample of real-world patients in the UK, using electronic health records (EHR).

*Methods:* From primary and secondary care EHR, we identified 85,732 patients with a HF diagnosis between 2002-2015. Almost 50% of HF patients were women and the median age was 79.1 [interquartile range 70.2-85.7] years, with age at diagnosis increasing over time.

*Results:* We found several trends in pharmacological HF management, including increased beta-blocker prescriptions over time (29% in 2002-2005 and 54% in 2013-2015), which was not observed for mineralocorticoid receptor-antagonists (MR-antagonists) (18% in 2002-2005 and 18% in 2013-2015); higher prescription rates of loop diuretics in women and elderly patients together with lower prescription rates of angiotensin converting enzyme-inhibitors and/or angiotensin II receptor blockers , beta-blockers, or MR-antagonists in these patients; and little change in medication prescription rates occurred after 6 months of HF diagnosis; and lastly, patients hospitalised for HF who had no recorded follow-up in primary care had considerably lower prescription rates compared to patients with a HF diagnosis in primary care with or without HF hospitalisation.

*Conclusion:* In the general population, the use of MR-antagonists for HF remained low and did not change throughout 13 years of follow up. For most patients, few changes were seen in pharmacological management of HF in the six months following diagnosis.


**Keywords:** Prescription patterns, ACE-inhibitors, ARB, RAS-inhibitors, Beta-blockers, MRA, Loop diuretics, Heart failure, Electronic health records.

**Article summary**



For peer review only - http://bmjopen.bmj.com/site/about/guidelines.xhtml

Strengths and limitations of the study

- Large cohort of HF patients from primary and secondary care
- Long follow-up period of almost 15 years
- Unable to differentiate between HF subphenotypes such as HF with reduced, mid-range or preserved ejection fraction.
- Unknown treatment eligibility, contraindications or intolerances that may affect the choice of treatment

For peer review only



For peer review only - http://bmjopen.bmj.com/site/about/guidelines.xhtml

**Abbreviations**

| | |
|---|---|
| HF | Heart failure |
| EHR | Electronic health records |
| CPRD | Clinical Practice Research Datalink |
| HES | Hospital Episode Statistics |
| ONS | Office for National Statistics |
| COPD | Chronic obstructive pulmonary disease |
| RAS-inhibitors | Angiotensin converting enzyme-inhibitors and/or angiotensin II receptor blockers |
| MR-antagonists | Mineralocorticoid receptor-antagonists |
| HFrEF | Heart failure with reduced ejection fraction |
| HFpEF | Heart failure with preserved ejection fraction |

For peer review only

## Introduction

Heart failure (HF) is a common public health burden, with the prevalence of HF estimated at approximately 500.000 patients in the UK.(1, 2) Once diagnosed, initiation and up titration of guideline recommended therapies can reduce morbidity and mortality, although 5-year survival still remains 20% to 50%.(3, 4)

Several observational studies have assessed treatment uptake in HF patients following their diagnosis. These studies suggest that many patients did not receive guideline recommended therapies, or at low doses with sparse attempts for up titration.(5–8) Optimal treatment for effective disease management seems to be particularly challenging in elderly patients, women or patients with multiple comorbidities and contraindications for treatments.(7, 8) At present, few data are available for prescription trends in HF patients in the general population and even fewer data are available that shed light on medication use in HF patients in the years prior to their HF diagnosis.

The CALIBER resource curates primary and secondary care EHR of 5 million individuals in the UK, including HF diagnosis and medication prescriptions.(9) Given the amount of information available, medication use of all HF patients in the community may be investigated – including those which are underrepresented in randomised clinical trials.

Therefore, we sought to examine HF treatment prescription patterns following a HF diagnosis for the overall population as well as specific subgroups based on gender (e.g. women), age (e.g. elderly), social economic status and healthcare setting (e.g. primary care or secondary care), in a large representative sample of real-world patients in the UK, using electronic health records (EHR). (10)

For peer review only



For peer review only - http://bmjopen.bmj.com/site/about/guidelines.xhtml

## Methods

*Data source*

Patients were selected from linked EHR in the UK, which consist of three linked databases: The Clinical Practice Research Datalink (CPRD) with primary care EHR, Hospital Episodes Statistics (HES) containing coded diagnoses and surgical procedures from inpatient hospital admissions, and the Office for National Statistics (ONS) registry containing cause-specific mortality data.(10) Previous work has shown that these patients are representative of the general population in the UK.(11, 12)

*Study population*

Patients were included at their first record of HF from CPRD or HES between January 1st 2002 and December 31st 2015. In CPRD, events were defined by a diagnosis of HF based on Read (version 2) controlled clinical terminology codes (NHS coded clinical terms) and in HES by a diagnosis of HF based on ICD-10 codes. The same HF diagnosis codes were used as in previous papers, with in addition several newer Read codes listed in **Table S1**.(4, 13) All patients were eligible for inclusion if they were aged 18 years or older, were registered with a GP for at least one year prior to diagnosis of HF, in a practice that had at least one year of up-to-standard data recording in CPRD (data quality check). The first record of HF from CPRD or HES was considered the index date. Individuals were censored at the earliest date from the date of de-registration in CPRD, the last data collection date of a practice in CPRD, the date of death or at the study end date (31st December 2015). Data on EHR phenotyping variables from HF patients up to 3 years prior to index date were included in this study.



For peer review only - http://bmjopen.bmj.com/site/about/guidelines.xhtml

*Patient and public involvement*

There was no patient or public involvement in this research.

*EHR phenotyping variables*

Baseline patient characteristics were based on records from CPRD and/or HES prior to index date, including demographics [age, sex, ethnicity, social deprivation] cardiovascular risk factors [smoking, BMI, diastolic blood pressure and systolic blood pressure and estimated glomerular filtration rate], comorbidities [a medical history of atrial fibrillation, chronic obstructive pulmonary disease (COPD), diabetes, hypertension, ischaemic heart disease, valvular disease and history of cancer] and medication prescription.

CPRD includes all prescriptions from the general practice. Prescriptions in CPRD were classified as: RAS-inhibitors (Angiotensin converting enzyme-inhibitors and/or angiotensin II receptor blockers), beta-blockers, mineralocorticoid receptor-antagonists (MR-antagonists) and loop diuretics. Definitions of these variables could be found online at http://www.caliberresearch.org/portal/.

Medication prescription for RAS-inhibitors, beta-blockers, MR-antagonists and loop diuretics was identified between three years prior to HF diagnosis up to three years after HF diagnosis per the following increments: -36 months to -24 months, -24 months to -18 months, -18 months to -12 months, -12 months to -6 months, -6 months to -3 months, -3 months to HF diagnosis, HF diagnosis to +3 months, +3 months to +6 months, +6 months to +12 months, +12 months to +18 months, +18 months to +24 months and +24 to +36 months.

Healthcare setting was characterised as primary care only (no HF hospitalisation), secondary care only (no Read HF diagnosis recorded in primary care) or HF diagnosis in both primary and secondary care. Ethnicity records from CPRD and HES were combined and



For peer review only - http://bmjopen.bmj.com/site/about/guidelines.xhtml

categorised as Caucasian, Asian, Black or Other. Social deprivation was measured as quintiles of the index of multiple deprivation of the geographical area of the primary care practice, a score calculated based on seven indices of deprivation: income, employment, health and disability, education, barrier to housing and services, crime and living environment.(14) Smoking status in CPRD was classified as never, ex- or current smokers.

*Statistical analysis*

Patient characteristics were summarised as mean (SD) or median [IQR] for continuous variables and percentages for categorical variables. The percentage of HF patients prescribed pharmacological treatments was calculated per increment and per time period as defined by publication year of previous ESC guidelines (2001, 2005, 2008 and 2012)(1, 15–18): 2002-2005, 2006-2008, 2009-2012 and 2013-2015. In addition to the overall cohort, we investigated several subgroups: age (< vs. ≥ 75 years old), sex (men vs. women), social economic status (lowest quintile of social deprivation vs. the rest) and setting (only follow-up in primary care vs. only in secondary care vs. follow-up in primary care after HF hospitalisation). All analyses were performed using R version 3.6.1.

For peer review only

## Results

*Baseline characteristics*

We identified 85,732 patients with a HF diagnosis. The study flow diagram could be found in **Figure S1**. Median follow-up after HF diagnosis (index date) was 2.1 years [0.6 – 4.5] years. **Table 1** shows the overall baseline patient characteristics and per time period 2002-2005, 2006-2008, 2009-2012 and 2013-2015. Almost 50% of patients were women and the median age was 79.1 [70.2 - 85.7] years, with age at HF diagnosis increasing over time. Overall, many



For peer review only - http://bmjopen.bmj.com/site/about/guidelines.xhtml

HF patients had comorbidities, most common were hypertension (61%), ischaemic heart disease (44%) and atrial fibrillation (37%), with increasing numbers of patients with comorbidities over time. Approximately 40% (n= 34,489) of patients were followed-up in primary care after a HF hospitalisation, 20% (n= 15,330) of patients were only known in primary care and never hospitalised for HF and the remaining 40% (n= 35,913) of patients had no follow-up in primary care after HF hospitalisation.

*Overall prescription patterns*

Overall prescription patterns are shown in **Figure 1**. Many patients were prescribed medication before HF diagnosis, especially RAS-inhibitors (20% in 2002-2005 to 46% in 2013-2015). Over time, beta-blocker prescription after HF diagnosis increased from 30% in 2002-2005 to 55% in 2013-2015. Throughout the follow up of 13 years, there were little observed changes for MR-antagonist uptake, this remained at 20% throughout time after HF diagnosis. The largest observed changes in prescription patterns occurred between 6 months before and after HF diagnosis (**Figure 1**). Approximately 20% of HF patients were prescribed a loop diuretic up to three years prior to HF diagnosis.

*Setting-specific prescription patterns*

Setting-specific prescription patterns are shown in **Figure 2**. Patients followed-up in primary care after HF hospitalisation had the highest prescription rates for all types of medication. Over time, the prescription for loop-diuretics, RAS-inhibitors and beta-blockers converged together. In these patients the prescription for MR-antagonists increased over time after HF diagnosis from 20% in 2002-2005 to 30% in 2013-2015.



For peer review only - http://bmjopen.bmj.com/site/about/guidelines.xhtml

Patients known in primary care but never hospitalised for HF had lower prescription rates for all types of treatment compared to patients with primary care follow-up and at least one HF hospitalisation. Mainly loop diuretics were less prescribed in these patients and the prescription of loop diuretics decreased over time with 65% of patients receiving loop diuretics after HF diagnosis in 2002-2005 compared to just over 40% in 2013-2015.

Patients hospitalised for HF but without a HF diagnosis in primary care, had the lowest prescriptions rates for loop diuretics, RAS-inhibitors and beta-blockers, which remained stable over time (50%, 45%, and 45% in 2013-2015 respectively). MR-antagonists were only prescribed in 13% of patients after HF diagnosis, this was similar for each time period.

*Age-specific prescription patterns*

Differences in prescription according to age categories are shown in **Figure 3**. The observed increase in prescriptions for RAS-inhibitors, beta-blockers, and MR-antagonists between 6 months before HF diagnosis to 6 months after HF diagnosis was less pronounced in elderly patients. The average increase in elderly patients was 12%, 7%, 8% for RAS-inhibitors, beta-blockers and MR-antagonists respectively, while younger patients had an average increase of 23%, 19% and 13% for RAS-inhibitors, beta-blockers and MR-antagonists respectively. On the other hand, a higher proportion of elderly patients were treated with loop-diuretics compared to younger patients, both before and after HF diagnosis (45% before and 63% after HF diagnosis in elderly compared to 27% before and 47% after HF diagnosis for younger patients in 2013-2015). After HF diagnosis, a higher percentage of younger patients were prescribed with RAS-inhibitors and beta-blockers compared to older patients.



For peer review only - http://bmjopen.bmj.com/site/about/guidelines.xhtml

*Sex-specific prescription patterns*

Differences in prescription between men and women are shown in **Figure 4**. Loop diuretics were prescribed in a higher proportion of women compared to men, this difference was already present prior to HF diagnosis where 6 months before diagnosis 30% of women and 20% of men were prescribed a loop diuretic. After HF diagnosis, the most prescribed medication for women was a loop diuretic, while a higher proportion of men were prescribed a RAS-inhibitor. Men were also more often prescribed RAS-inhibitors, beta-blockers and MR-antagonists after HF diagnosis compared to women.

*Social economic status-specific prescription patterns*

Social economic status-specific prescription patterns are shown in **Figure 5**. We did not observe any discernible differences between patients in low vs. high social-economic areas (highest quintile of social economic deprivation).

## Discussion

In this large-scale study of 85,732 HF patients we investigated treatment prescription patterns in a representative sample of real-world patients with HF in the UK between 2002 and 2015. We found three important trends in pharmacological HF management: a) increased use of beta-blockers, whereas there was no increased uptake of MR-antagonists over 13 years follow up; b) prescription rates remained almost unchanged after the first 6 months following a HF diagnosis; and lastly, c) higher rates of loop diuretics in women and elderly patients together with lower prescription rates for RAS-inhibitors, beta-blockers, or MR-antagonists.

For peer review only

*Temporal trends in heart failure medication*

Even though prescription rates increased over time from 2002 to 2015, overall prescription rates remained low. This is in line with previously published studies.(5–8, 19) Low prescription rates could be attributed to the mixed HF cases found in EHR. We were unable to distinguish HF with reduced ejection fraction (HFrEF), HF with mid-range ejection fraction, and HF with preserved ejection fraction (HFpEF) based on medical records, thereby including known differences in treatment recommendations for these HF phenotypes.(1)

We found no major differences in prescription behaviour after the publication of ESC guidelines, however we did observe the gradual increase of beta-blockers as one of the cornerstones of HF treatment. RAS-inhibitors were prescribed in a high proportion of patients throughout the years of the study, presumably because the first clinical trials in HFrEF showing a beneficial effect were from the late 1980s and early 1990s.(20) Surprisingly, we found lower than expected prescription rates for MR-antagonists, which persisted over the years included in this study. This is in spite of multiple clinical trials which have shown benefit in HFrEF patients.(21) Besides HFrEF trials, a post-hoc analysis of the TOPCAT trial in 2015 (Spironolactone, a MR-antagonist, for HFpEF) reported regional differences between Americas and Russia/Georgia, where the American patients showed clinical benefits.(22) The American College of Cardiology/American Heart Association focused update on HF management in 2017 gave spironolactone a grade IIb recommendation, thereby stimulating that selected HFpEF patients could be treated with spironolactone to decrease re-hospitalisations.(23)

*Heart failure medication initiation following diagnosis*

Most activity in treatment prescription behaviour was observed between 6 months before to 6 months after HF diagnosis. After the 6 month mark we did not observe many patients starting



For peer review only - http://bmjopen.bmj.com/site/about/guidelines.xhtml

any of the medication investigated. This is in line with previous studies showing that there are few changes in medication use and little up titration of medication after treatment initiation.(5, 24) This leaves room for improvement in starting treatment at any time point following a HF diagnosis, for example if patients hospitalised with acute HF do not immediately tolerate negative inotropic medication such as beta-blockers.

*Impact of heart failure hospitalisation on medication prescription*

We found differences in prescription patterns between patients with a HF diagnosis recorded in different settings. Patients with a primary care HF diagnosis without HF hospitalisation had much lower prescription rates of loop diuretics compared to patients with a HF diagnosis recorded in both primary and secondary care. It could be that these patients have less severe fluid overload or symptoms that requires alleviation by loop diuretics, and thus less severe HF.

Previously it was shown that there are differences in overall five-year survival of patients with HF diagnosis recorded in primary care only, secondary care only and in both, with the worst survival seen in HF patients identified only in secondary care and the best survival for HF patients identified in primary care with or without hospitalisation for HF.(4) Here, we advance current knowledge by showing that there are longitudinal differences in HF care of patients with diagnosis recorded in different settings.

In this study almost 40% of patients did not have a GP record of a HF diagnosis after a HF hospitalisation. One reason could be that GPs do not formally register HF with a Read diagnosis code, but rather in free text descriptions. However, there could also be a potential quality of care gap or failure of communication between secondary and primary care, where patients are not treated optimally. Primary care is the basis of many healthcare systems, including the UK. If there is no HF diagnosis recorded in primary care after HF hospitalisation,



For peer review only - http://bmjopen.bmj.com/site/about/guidelines.xhtml

For peer review only

which is shown to indicative for worse survival, rehospitalisation and severity of disease, this could be detrimental for patients.

*Heart failure treatment in women and elderly*

Over time, we observed that HF was diagnosed at a later age, with the median almost 80 years old between 2013-2015. This is also seen in many other developed countries where the mean age of HF diagnosis is over 70 years old.(25, 26)

We observed lower prescription rates in elderly patients compared to younger patients for RAS-inhibitors, beta-blockers and MR-antagonists, although the difference in MR-antagonists was less pronounced. Many elderly patients were already using RAS-inhibitors prior to HF diagnosis, therefore the increase in prescription rate is not as steep as compared to younger HF patients who are prescribed less medication prior to HF diagnosis. This could be explained by the presence of comorbidities, such as atrial fibrillation or hypertension, which are much more prevalent among elderly compared to younger patients, and for which these elderly patients could be prescribed RAS-inhibitors.

Remarkably, the difference between prescription of RAS-inhibitors and beta-blockers prior to HF diagnosis was less than 5% for men and women, and only after the diagnosis of HF was a higher proportion of men prescribed a RAS-inhibitor or beta-blocker. This could potentially be related to the fact that elderly women are more likely to develop HFpEF and therefore tend to be treated symptomatically with loop diuretics, rather than with RAS-inhibitors and beta-blockers. However, the literature also shows that there are differences in treatment prescription in men and women with HFrEF, for which there is no obvious explanation.(27)



For peer review only - http://bmjopen.bmj.com/site/about/guidelines.xhtml

Both elderly patients and women received more loop diuretics. However, this could potentially be harmful, especially for elderly, since loop diuretics could lead to electrolyte disturbances and acute kidney injury.(28) Elderly patients are often excluded or underrepresented in clinical trials, therefore current recommendations lack convincing evidence in the elderly population. However, recently a large meta-analysis reported a significant effect of beta-blockers on overall mortality regardless of age.(29) These studies indicate that elderly patients also benefit from HF-specific medication and should be a choice of treatment for these patients, besides loop diuretics for symptom alleviation. However, elderly patients might have more contraindications or intolerances to RAS-inhibitors, beta-blockers and MR-antagonists and might therefore be more often treated with loop diuretics for symptom control.

*Strengths and limitations*

Strengths of this study are the large cohort of HF patients and a long follow-up period. Patient records available are representative of the general UK population, which provides evidence for the validity of using these EHR for research.(11, 12) However, we were limited by the inability to differentiate between HF phenotypes based on medical records, since there was no access to detailed echocardiography estimates to assess systolic function. Nor did we have information on NYHA class or NT-proBNP biomarker levels. Furthermore, treatments administered during a hospital admission or discharge were not reported, such as intravenous inotropic agents. However, CPRD includes all prescriptions from general practice to non-hospitalized patients. We were also unable to assess patients' symptom class (which would affect their eligibility for treatments such as MRA-antagonists), and contraindications or intolerances that may affect the choice of medication.



For peer review only - http://bmjopen.bmj.com/site/about/guidelines.xhtml

*Conclusion*

The results of this population-based study of over 80,000 patients with heart failure in England shows variable increases in uptake of evidence-based treatments, with no change in prescription of MR-antagonists over 13 years, but an increase in beta-blocker use. Large differences were observed between HF patient groups, with lowest prescription rates of RAS-inhibitors, beta-blockers and MR-antagonists in women, elderly patients, and those without a HF diagnosis in primary care. Most changes in prescriptions occurred within 6 months prior to or 6 months following a diagnosis of HF, with little change thereafter, suggesting further opportunities to improve HF management.



For peer review only - http://bmjopen.bmj.com/site/about/guidelines.xhtml

**Acknowledgements**

This study was approved by the Medicines and Healthcare Products Regulatory Agency (UK) Independent Scientific Advisory Committee protocol reference: 17_015, under Section 251 (NHS Social Care Act 2006). This study is based in part on data from the Clinical Practice Research Datalink obtained under license from the UK Medicines and Healthcare products Regulatory Agency. The data are provided by patients and collected by the NHS as part of their care and support. The interpretation and conclusions contained in this study are those of the author(s) alone. Hospital Episode Statistics Copyright (2019) is reused with the permission of The Health & Social Care Information Centre. All rights reserved. "This study was carried out as part of the CALIBER © resource (https://www.ucl.ac.uk/health-informatics/caliber and https://www.caliberresearch.org/). CALIBER, led from the UCL Institute of Health Informatics, is a research resource providing validated electronic health record phenotyping algorithms and tools for national structured data sources.

**a. Contributor statement**

AU has designed the research, analysed and interpreted the data and drafted the manuscript. IV, AH, FA and SK have designed the research, interpreted the data, critically revised the manuscript and supervised AU. SD, HH, AS, JC and DG have interpreted the data and critically revised the manuscript.

**b. Competing interests**

The authors report no conflict of interest.



For peer review only - http://bmjopen.bmj.com/site/about/guidelines.xhtml

### c. Funding

This study is part of the BigData@Heart program that has received funding from the Innovative Medicines Initiative 2 Joint Undertaking under grant agreement No 116074. This Joint Undertaking receives support from the European Union's Horizon 2020 research and innovation programme and EFPIA.

This work was supported by Health Data Research UK [grant number N/A], which receives its funding from Health Data Research UK Ltd (NIWA1) funded by the UK Medical Research Council, Engineering and Physical Sciences Research Council, Economic and Social Research Council, Department of Health and Social Care (England), Chief Scientist Office of the Scottish Government Health and Social Care Directorates, Health and Social Care Research and Development Division (Welsh Government), Public Health Agency (Northern Ireland), British Heart Foundation, and the Wellcome Trust. Work at the University College London Institute of Health Informatics and Institute of Cardiovascular Science is supported by a British Heart Foundation Accelerator Award (AA/18/6/24223).

**FA** is supported by UCL Hospitals NIHR Biomedical Research Centre. **IV** is supported by a grant from the Dutch Heart Foundation [grant DHF project 'Facts and Figures']. **SD** is supported by an Alan Turing Fellowship. **HH** is supported by an NIHR Senior Investigator Award. **ADS** is funded by a post-doctoral fellowship from THIS Institute. **JC** received research grants from Bayer, Novartis and Vifor and honoraria for steering committees from Amgen, Bayer, Novartis and Servier.

For peer review only

**d. Data sharing statement**

All data were provided anonymised and are not publicly available due to their sensitive nature. Data may be obtained from the Clinical Practice Research Datalink (https://www.cprd.com). EHR phenotypes are available from the CALIBER resource (https://www.caliberresearch.org). The protocol may be obtained via the Clinical Practice Research Datalink under protocol reference: 17_015. No additional data is available.

For peer review only

## References

1. Ponikowski P, Voors AA, Anker SD, et al. 2016 ESC Guidelines for the diagnosis and treatment of acute and chronic heart failure: The Task Force for the diagnosis and treatment of acute and chronic heart failure of the European Society of Cardiology (ESC). Developed with the special contribution . Eur. J. Heart Fail. 2016;18:891–975.

2. British Heart Foundation Centre on Population Approaches for Non-Communicable Disease Prevention. British Heart Foundation Cardiovascular Disease Statistics 2015. 2015.

3. Bleumink GS, Knetsch AM, Sturkenboom MCJM, et al. Quantifying the heart failure epidemic: prevalence, incidence rate, lifetime risk and prognosis of heart failure The Rotterdam Study. Eur. Heart J. 2004;25:1614–1619.

4. Koudstaal S, Pujades-Rodriguez M, Denaxas S, et al. Prognostic burden of heart failure recorded in primary care, acute hospital admissions, or both: a population-based linked electronic health record cohort study in 2.1 million people. Eur. J. Heart Fail. 2017;19:1119-1127.

5. Deschaseaux C, McSharry M, Hudson E, Agrawal R, Turner SJ. Treatment Initiation Patterns, Modifications, and Medication Adherence Among Newly Diagnosed Heart Failure Patients: A Retrospective Claims Database Analysis. J. Manag. care Spec. Pharm. 2016;22:561–571.

6. Bress AP, King JB, Brixner D, et al. Pharmacotherapy Treatment Patterns, Outcomes, and Health Resource Utilization Among Patients with Heart Failure with Reduced Ejection Fraction at a U.S. Academic Medical Center. Pharmacotherapy 2016;36:174–186.

7. Stork S, Handrock R, Jacob J, et al. Treatment of chronic heart failure in Germany: a retrospective database study. Clin. Res. Cardiol. 2017;106:923-932.

8. Chin KL, Skiba M, Tonkin A, et al. The treatment gap in patients with chronic systolic heart failure: a systematic review of evidence-based prescribing in practice. Heart Fail. Rev.



For peer review only - http://bmjopen.bmj.com/site/about/guidelines.xhtml

2016;21:675–697.

9. Denaxas S, Gonzalez-Izquierdo A, Direk K, et al. UK phenomics platform for developing and validating electronic health record phenotypes: CALIBER. J. Am. Med. Inform. Assoc. 2019;26:1545-1559.

10. Denaxas SC, George J, Herrett E, et al. Data resource profile: cardiovascular disease research using linked bespoke studies and electronic health records (CALIBER). Int. J. Epidemiol. 2012;41:1625–1638.

11. Herrett E, Thomas SL, Schoonen WM, Smeeth L, Hall AJ. Validation and validity of diagnoses in the General Practice Research Database: a systematic review. Br. J. Clin. Pharmacol. 2010;69:4–14.

12. Burns EM, Rigby E, Mamidanna R, et al. Systematic review of discharge coding accuracy. J. Public Health. 2012;34:138–148.

13. Uijl A, Koudstaal S, Direk K, et al. Risk factors for incident heart failure in age- and sex-specific strata: a population-based cohort using linked electronic health records. Eur. J. Heart Fail. 2019;21:1197-1206.

14. McLennan D, Barnes H, Noble M, Davies J, Garratt E. The English indices of deprivation, 2010: Technical Report. London: HMSO. 2011.

15. Remme WJ, Swedberg K. Guidelines for the diagnosis and treatment of chronic heart failure. Eur. Heart J. 2001;22:1527–1560.

16. Swedberg K, Drexler H, Follath F, et al. Guidelines for the diagnosis and treatment of chronic heart failure: executive summary ( update 2005 ) The Task Force for the Diagnosis and Treatment of Chronic Heart Failure of the European Society of Cardiology. Eur. Heart J. 2005:1115–1140.

17. Dickstein K, Cohen-Solal A, Filippatos G, et al. ESC guidelines for the diagnosis and



For peer review only - http://bmjopen.bmj.com/site/about/guidelines.xhtml

For peer review only

treatment of acute and chronic heart failure 2008: the Task Force for the diagnosis and treatment of acute and chronic heart failure 2008 of the European Society of Cardiology. Developed in collaboration with the Heart. Eur. J. Heart Fail. 2008;10:933–989.

18. McMurray JJV, Adamopoulos S, Anker SD, et al. ESC Guidelines for the diagnosis and treatment of acute and chronic heart failure 2012: The Task Force for the Diagnosis and Treatment of Acute and Chronic Heart Failure 2012 of the European Society of Cardiology. Developed in collaboration with the Heart Failure Association (HFA) of the ESC. Eur. Heart J. 2012;33:1787–1847.

19. Peters-Klimm F, Muller-Tasch T, Schellberg D, et al. Guideline adherence for pharmacotherapy of chronic systolic heart failure in general practice: a closer look on evidence-based therapy. Clin. Res. Cardiol. 2008;97:244–252.

20. SOLVD Investigators, Yusuf S, Pitt B, Davis CE, Hood WB, Cohn JN. Effect of enalapril on survival in patients with reduced left ventricular ejection fractions and congestive heart failure. N. Engl. J. Med. 1991;325:293–302.

21. Pitt B, Zannad F, Remme WJ, et al. The Effect of Spironolactone on Morbidity and Mortality in Patients with Severe Heart Failure. N. Engl. J. Med. 1999;341:709–717.

22. Pfeffer MA, Claggett B, Assmann SF, et al. Regional Variation in Patients and Outcomes in the Treatment of Preserved Cardiac Function Heart Failure With an Aldosterone Antagonist (TOPCAT) Trial. Circulation 2015;131:34–42.

23. Yancy CW, Jessup M, Bozkurt B, et al. 2017 ACC/AHA/HFSA Focused Update of the 2013 ACCF/AHA Guideline for the Management of Heart Failure: A Report of the American College of Cardiology/American Heart Association Task Force on Clinical Practice Guidelines and the Heart Failure Society of Amer. J. Am. Coll. Cardiol. 2017;68:1476–1488.

24. Greene SJ, Fonarow GC, DeVore AD, et al. Titration of Medical Therapy for Heart Failure



For peer review only - http://bmjopen.bmj.com/site/about/guidelines.xhtml

With Reduced Ejection Fraction. J. Am. Coll. Cardiol. 2019;73:2365–2383.

25. Brugts JJ, Linssen GCM, Hoes AW, Brunner-La Rocca HP. Real-world heart failure management in 10,910 patients with chronic heart failure in the Netherlands. Netherlands Hear. J. 2018;26:272–279.

26. Savarese G, Vasko P, Jonsson Å, Edner M, Dahlström U, Lund LH. The Swedish Heart Failure Registry: a living, ongoing quality assurance and research in heart failure. Ups. J. Med. Sci. 2019;124:65–69.

27. Dewan P, Rørth R, Jhund PS, et al. Differential Impact of Heart Failure With Reduced Ejection Fraction on Men and Women. J. Am. Coll. Cardiol. 2019;73:29–40.

28. Täger T, Fröhlich H, Seiz M, Katus HA, Frankenstein L. READY: relative efficacy of loop diuretics in patients with chronic systolic heart failure—a systematic review and network meta-analysis of randomised trials. Heart Fail. Rev. 2019;24:461-472.

29. Kotecha D, Manzano L, Krum H, et al. Effect of age and sex on efficacy and tolerability of beta blockers in patients with heart failure with reduced ejection fraction: individual patient data meta-analysis. BMJ 2016;353:i1855.

For peer review only



For peer review only - http://bmjopen.bmj.com/site/about/guidelines.xhtml

**Figure legends**

**Central illustration Figure 1**

**Legend Figure 1.** The percentage of HF patients receiving prescriptions of RAS-inhibitors, beta-blockers, MR-antagonists, loop diuretics per months since HF diagnosis. RAS-inhibitors = ACE-inhibitors and/or ARB; MR-antagonists = mineralocorticoid receptor antagonists.

**Legend Figure 2.** The percentage of HF patients receiving prescriptions of RAS-inhibitors, beta-blockers and MRAs per months since HF diagnosis, stratified by setting (primary care only, secondary care only, both primary and secondary care). RAS-inhibitors = ACE-inhibitors and/or ARB; MR-antagonists = mineralocorticoid receptor antagonists.

**Legend Figure 3.** The percentage of HF patients receiving prescriptions of RAS-inhibitors, beta-blockers and MRAs per months since HF diagnosis, stratified by age. RAS-inhibitors = ACE-inhibitors and/or ARB; MR-antagonists = mineralocorticoid receptor antagonists.

**Legend Figure 4.** The percentage of HF patients receiving prescriptions of RAS-inhibitors, beta-blockers and MRAs per months since HF diagnosis, stratified by sex. RAS-inhibitors = ACE-inhibitors and/or ARB; MR-antagonists = mineralocorticoid receptor antagonists.

**Legend Figure 5.** The percentage of HF patients receiving prescriptions of RAS-inhibitors, beta-blockers and MRAs per months since HF diagnosis, stratified by social status (highest quintile of social deprivation vs. the rest). RAS-inhibitors = ACE-inhibitors and/or ARB; MR-antagonists = mineralocorticoid receptor antagonists.

For peer review only

**Table 1. Patients characteristics of heart failure patients between 2002 and 2015**

| | Overall | 2002 - 2005 | 2006 - 2008 | 2009 - 2012 | 2013 - 2015 | % missing |
|---|---|---|---|---|---|---|
| n | 85732 | 25366 | 17715 | 26114 | 16537 | |
| **Demographics** | | | | | | |
| Age (Years, median [IQR]) | 79.1 [70.2, 85.7] | 78.7 [70.7, 84.9] | 78.7 [ 69.9, 85.4] | 79.5 [70.1, 86.3] | 79.7 [70.0, 86.4] | 0 |
| Sex (% Women) | 48.6 | 49.3 | 48.4 | 48.4 | 48.0 | 0 |
| Ethnicity (% Caucasian) | 96.5 | 97.5 | 96.9 | 96.1 | 95.1 | 3.5 |
| Social deprivation (% lowest quintile) | 24.3 | 25.1 | 25.0 | 24.0 | 22.9 | 0 |
| **Clinical and lifestyle measurements** | | | | | | |
| SBP (mmHg, mean (sd)) | 136.2 (20.7) | 140.6 (22.3) | 135.9 (20.7) | 134.6 (20.0) | 132.9 (18.7) | 13.0 |
| DBP (mmHg, mean (sd)) | 76.2 (12.0) | 78.4 (12.0) | 76.2 (12.0) | 75.4 (12.0) | 74.4 (11.6) | 13.0 |
| BMI (kg/$m^2$, mean (sd)) | 28.6 (6.6) | 28.2 (6.4) | 28.4 (6.6) | 28.7 (6.8) | 28.8 (6.8) | 54.0 |
| eGFR (min/m2/1.73mL, median [IQR]) | 58.4 [ 45.3, 72.1] | 54.7 [43.4, 66.1] | 56.5 [44.3, 68.8] | 60.5 [46.3, 75.3] | 62.9 [47.5, 78.2] | 24.0 |
| Smoking status (% Current) | 20.8 | 22.3 | 20.0 | 20.4 | 20.5 | 38.7 |
| **Medical history (%)**[¶] | | | | | | |
| Atrial Fibrillation | 36.6 | 28.4 | 36.3 | 40.6 | 43.0 | - |
| COPD | 17.9 | 14.8 | 17.3 | 19.5 | 21.0 | - |
| Diabetes | 22.3 | 18.1 | 22.2 | 23.7 | 26.7 | - |
| Hypertension | 60.7 | 46.0 | 60.7 | 67.9 | 72.0 | - |
| Ischaemic heart disease | 44.2 | 39.0 | 46.0 | 46.4 | 46.8 | - |
| Valvular disease | 16.5 | 9.5 | 14.9 | 19.9 | 23.8 | - |
| **Medication prescription up to 3 months after HF diagnosis (%)**[¶] | | | | | | |
| RAS-inhibitors | 60.8 | 59.6 | 63.5 | 62.0 | 57.6 | - |
| Beta-blockers | 42.5 | 28.9 | 41.0 | 49.3 | 54.1 | - |
| MR-antagonists | 18.0 | 18.4 | 17.9 | 17.6 | 18.2 | - |
| Loop diuretics | 63.0 | 68.4 | 63.5 | 61.1 | 57.0 | - |

For peer review only

**Legend Table 1.** * Assessed by index of multiple deprivation, § denotes prior medical history of given comorbidity, ¤ Medication 12 months prior to index date, Mean (SD) = Mean (Standard deviation), Median [IQR] = Median [Interquartile range], CPRD = Clinical Practice Research Datalink, SBP = systolic blood pressure, DBP = Diastolic blood pressure, BMI = Body Mass Index, eGFR = estimated glomerular filtration rate, COPD = Chronic Obstructive Pulmonary Disease, RAS-inhibitors = ACE-inhibitors and/or angiotensin II receptor blockers, MR-antagonists = mineralocorticoid receptor antagonist. ¶Medical conditions and prescriptions were considered absent if not recorded.

For peer review only

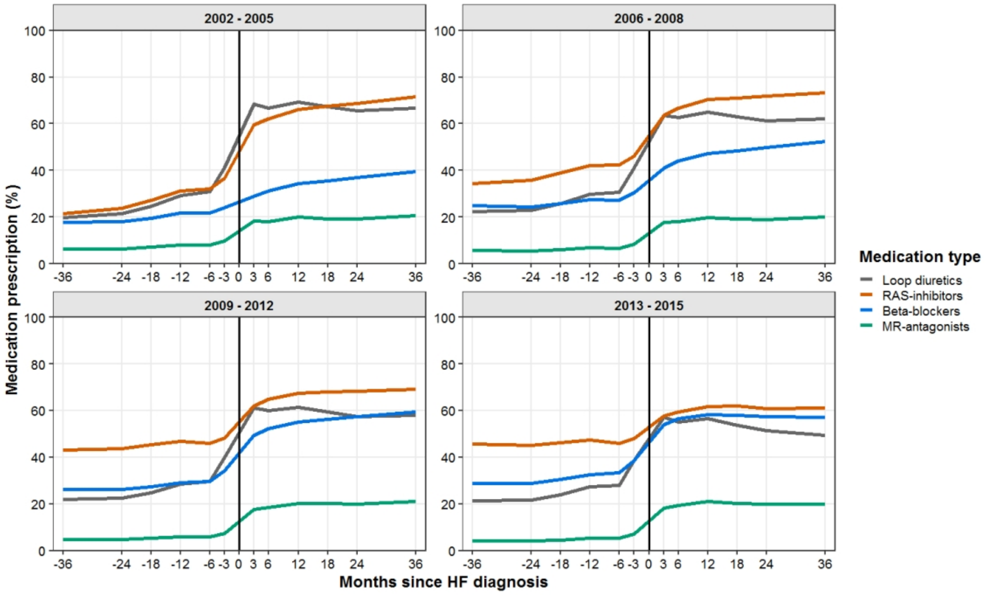


figure 1

127x76mm (300 x 300 DPI)

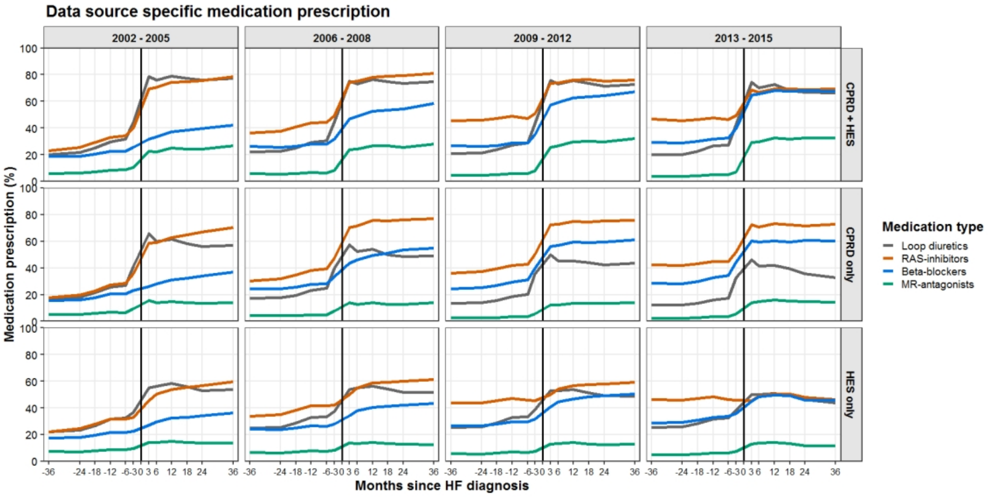


figure 2

152x76mm (300 x 300 DPI)

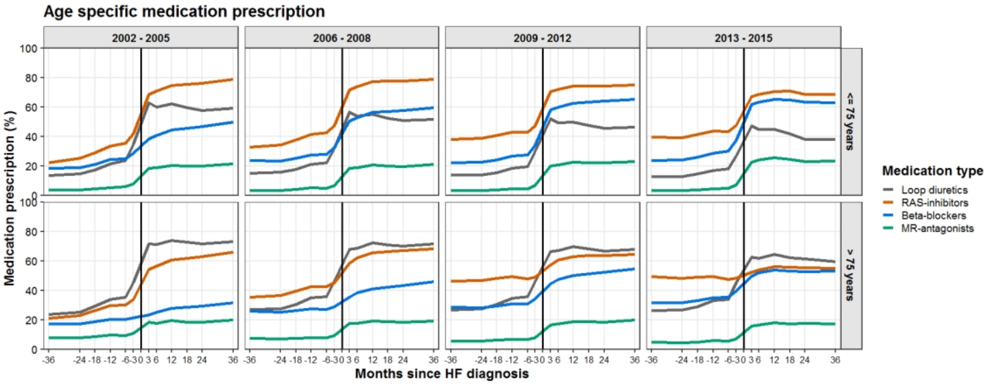


figure 3

152x59mm (300 x 300 DPI)

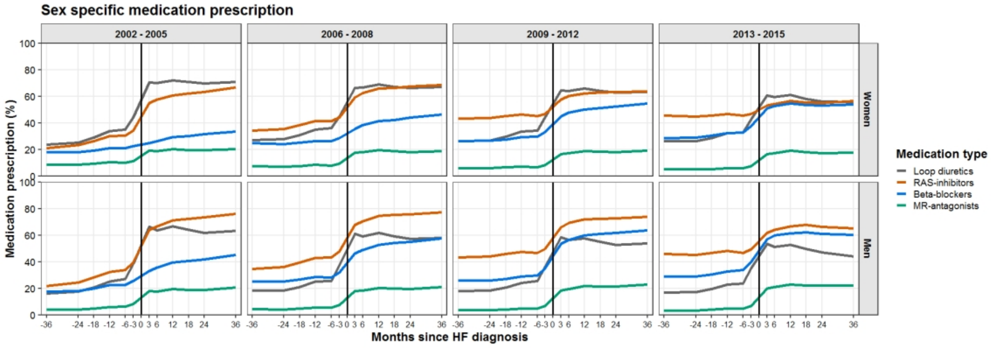


figure 4

169x59mm (300 x 300 DPI)

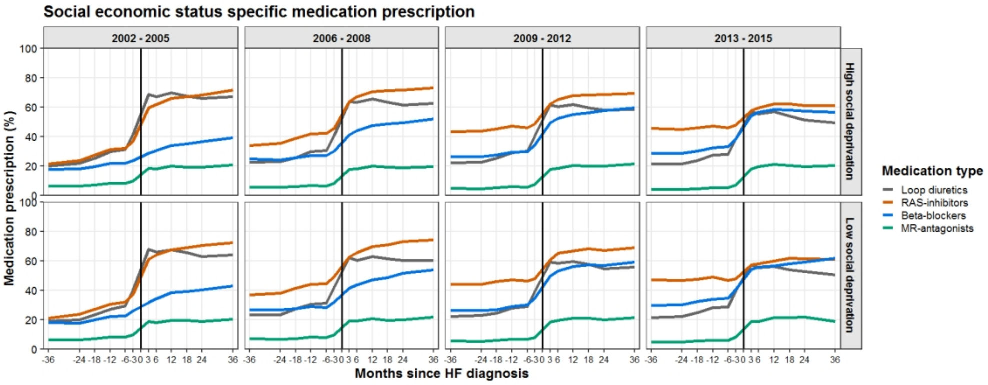


figure 5

152x59mm (300 x 300 DPI)

**Supplemental material**

**Figure S1 – study flow diagram**

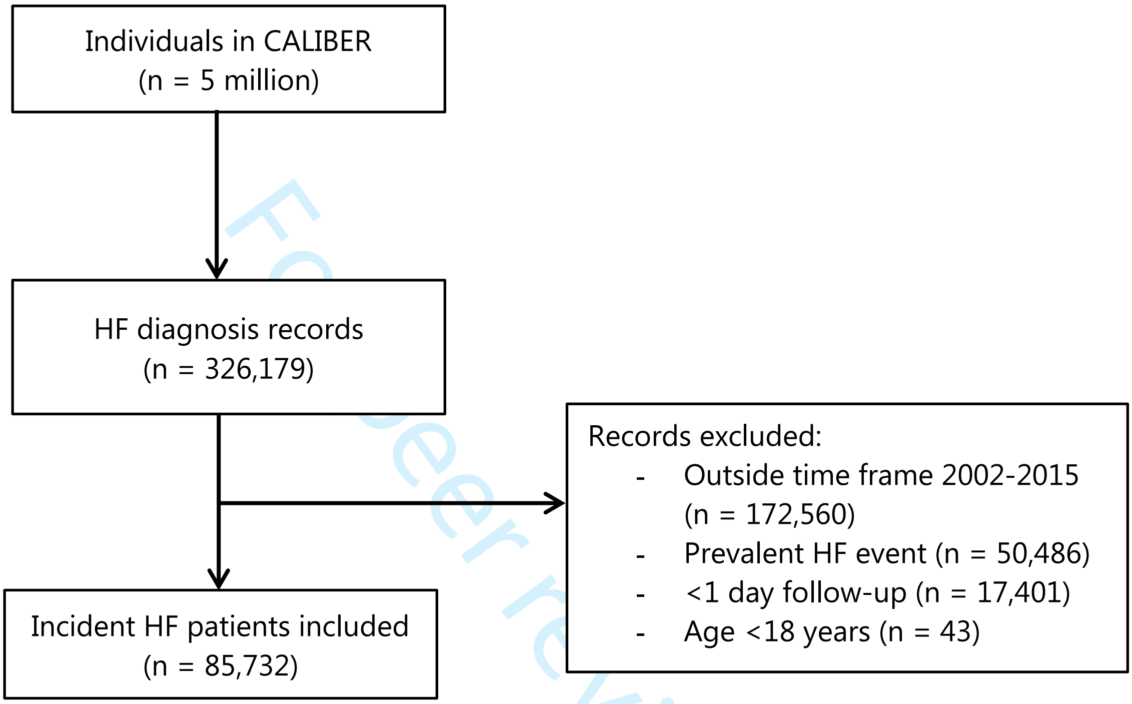


For peer review only

**Table S1 – additional READ codes used to identify heart failure in the Clinical Practice Research Datalink**

| CPRD* Heart Failure READ codes |
| --- |
| 585g.00, G5yyC00, G5yyA00, G583.12, G583.11, G583.00, G5yy900, 585f.00 |

**Legend Table S1**. * CPRD = Clinical Practice Research Datalink

For peer review only

STROBE Statement—Checklist of items that should be included in reports of ***cohort studies***

| | Item No | Recommendation | Page No |
|---|---|---|---|
| **Title and abstract** | 1 | (*a*) Indicate the study's design with a commonly used term in the title or the abstract<br>(*b*) Provide in the abstract an informative and balanced summary of what was done and what was found | 1, 3 |
| **Introduction** | | | |
| Background/rationale | 2 | Explain the scientific background and rationale for the investigation being reported | 6 |
| Objectives | 3 | State specific objectives, including any prespecified hypotheses | 6 |
| **Methods** | | | |
| Study design | 4 | Present key elements of study design early in the paper | 7 |
| Setting | 5 | Describe the setting, locations, and relevant dates, including periods of recruitment, exposure, follow-up, and data collection | 7 |
| Participants | 6 | (*a*) Give the eligibility criteria, and the sources and methods of selection of participants. Describe methods of follow-up<br>(*b*) For matched studies, give matching criteria and number of exposed and unexposed | 7 |
| Variables | 7 | Clearly define all outcomes, exposures, predictors, potential confounders, and effect modifiers. Give diagnostic criteria, if applicable | 8 |
| Data sources/ measurement | 8* | For each variable of interest, give sources of data and details of methods of assessment (measurement). Describe comparability of assessment methods if there is more than one group | - |
| Bias | 9 | Describe any efforts to address potential sources of bias | - |
| Study size | 10 | Explain how the study size was arrived at | - |
| Quantitative variables | 11 | Explain how quantitative variables were handled in the analyses. If applicable, describe which groupings were chosen and why | - |
| Statistical methods | 12 | (*a*) Describe all statistical methods, including those used to control for confounding<br>(*b*) Describe any methods used to examine subgroups and interactions<br>(*c*) Explain how missing data were addressed<br>(*d*) If applicable, explain how loss to follow-up was addressed<br>(*e*) Describe any sensitivity analyses | 9 |
| **Results** | | | |
| Participants | 13* | (a) Report numbers of individuals at each stage of study—eg numbers potentially eligible, examined for eligibility, confirmed eligible, included in the study, completing follow-up, and analysed<br>(b) Give reasons for non-participation at each stage<br>(c) Consider use of a flow diagram | 9 |
| Descriptive data | 14* | (a) Give characteristics of study participants (eg demographic, clinical, social) and information on exposures and potential confounders<br>(b) Indicate number of participants with missing data for each variable of interest<br>(c) Summarise follow-up time (eg, average and total amount) | 9 - 10 |
| Outcome data | 15* | Report numbers of outcome events or summary measures over time | 10 - 12 |

For peer review only


For peer review only - http://bmjopen.bmj.com/site/about/guidelines.xhtml

| | | | |
|---|---|---|---|
| Main results | 16 | (*a*) Give unadjusted estimates and, if applicable, confounder-adjusted estimates and their precision (eg, 95% confidence interval). Make clear which confounders were adjusted for and why they were included<br>(*b*) Report category boundaries when continuous variables were categorized<br>(*c*) If relevant, consider translating estimates of relative risk into absolute risk for a meaningful time period | 10 - 12 |
| Other analyses | 17 | Report other analyses done—eg analyses of subgroups and interactions, and sensitivity analyses | - |
| **Discussion** | | | |
| Key results | 18 | Summarise key results with reference to study objectives | 12 |
| Limitations | 19 | Discuss limitations of the study, taking into account sources of potential bias or imprecision. Discuss both direction and magnitude of any potential bias | 16 |
| Interpretation | 20 | Give a cautious overall interpretation of results considering objectives, limitations, multiplicity of analyses, results from similar studies, and other relevant evidence | 13 - 15 |
| Generalisability | 21 | Discuss the generalisability (external validity) of the study results | 13 - 15 |
| **Other information** | | | |
| Funding | 22 | Give the source of funding and the role of the funders for the present study and, if applicable, for the original study on which the present article is based | 2 |

*Give information separately for exposed and unexposed groups.

**Note:** An Explanation and Elaboration article discusses each checklist item and gives methodological background and published examples of transparent reporting. The STROBE checklist is best used in conjunction with this article (freely available on the Web sites of PLoS Medicine at http://www.plosmedicine.org/, Annals of Internal Medicine at http://www.annals.org/, and Epidemiology at http://www.epidem.com/). Information on the STROBE Initiative is available at http://www.strobe-statement.org.

For peer review only
